# The American Association of Tissue Banks tissue donor screening for *Mycobacterium tuberculosis*—Recommended criteria and literature review

**DOI:** 10.1111/tid.14294

**Published:** 2024-06-09

**Authors:** Melissa A. Greenwald, Noelle Edwards, D. Ted Eastlund, Inga Gurevich, Andrea Pervine‐Zaman Ho, Ghada Khalife, Janet Lin‐Torre, Hannis W. Thompson, Ross M Wilkins, Sally F. Alrabaa

**Affiliations:** ^1^ American Association of Tissue Banks McLean Virginia USA; ^2^ Uniformed Services University Bethesda Maryland USA; ^3^ Donor Alliance Denver Colorado USA; ^4^ LifeNet Health Virginia Beach Virginia USA; ^5^ University of New Mexico Albuquerque New Mexico USA; ^6^ AlloSource Centennial Colorado USA; ^7^ Solvita Dayton Ohio USA; ^8^ Wright State University Dayton Ohio USA; ^9^ MTF Biologics Edison New Jersey USA; ^10^ Department of Medicine Cooperman Barnabas Medical Center Livingston New Jersey USA; ^11^ University of South Florida Morsani College of Medicine Tampa Florida USA; ^12^ LifeLink Tissue Bank Tampa Florida USA

**Keywords:** donor eligibility, donor screening, exclusion criteria, tissue transmission, tissue transplantation, tuberculosis

## Abstract

After two multistate outbreaks of allograft tissue‐transmitted tuberculosis (TB) due to viable bone, evidence‐based donor screening criteria were developed to decrease the risk of transmission to recipients. Exclusionary criteria, commentary, and references supporting the criteria are provided, based on literature search and expert opinion. Both exposure and reactivation risk factors were considered, either for absolute exclusion or for exclusion in combination with multiple risk factors. A criteria subset was devised for tissues containing viable cells. Risk factors for consideration included exposure (e.g., geographic birth and residence, travel, homelessness, incarceration, healthcare, and workplace) and reactivation (e.g., kidney disease, liver disease, history of transplantation, immunosuppressive medications, and age). Additional donor considerations include the possibility of sepsis and chronic illness. Donor screening criteria represent minimal criteria for exclusion and do not completely exclude all possible donor TB risks. Additional measures to reduce transmission risk, such as donor and product testing, are discussed but not included in the recommendations. Careful donor evaluation is critical to tissue safety.

## 1 INTRODUCTION

Tuberculosis (TB), caused by *Mycobacterium tuberculosis* (MTB), is one of the most common infectious diseases worldwide, with high morbidity and mortality. Historically, tissue transmission of MTB has been rarely reported, but transmission was recently documented via bone marrow/matrix tissue allograft in 2021^1^ and 2023.^2^ This allograft product is a mixture of bone matrix materials and cryopreserved marrow elements from a single donor used to promote bone healing and spinal fusion. After notification of the first transmission investigation, the American Association of Tissue Banks (AATB) Physicians Council formed an MTB working group (WG) to review the literature and evaluate donor screening criteria for tissue donors with respect to MTB. This work has resulted in multiple updates to the tissue transplant community since 2021^3^, ^4^, ^5^ including interim MTB tissue donor screening requirements for AATB members to address urgent public safety needs. The WG has developed comprehensive donor screening criteria and this literature review is the first known review of MTB from the perspective of tissue donor screening.

Tissue transplantation has a low rate of communicable disease transmission, with millions of allografts transplanted annually.^6^ Available data indicate that transmission of infections occurs less frequently than in organ transplantation, but more frequently than via blood transfusion.^7^ Historically tissue‐transmitted infections are uncommon; those reported are largely due to fastidious bacteria such as *Clostridium* ^8^ and group *A Streptococcus* species.^9^ Allografts with minimal processing, such as tendons, tendons with bone, saphenous veins, cardiopulmonary patch, and non‐irradiated bone have a higher rate of communicable disease transmission than highly processed tissues.^10^, ^11^ Given these data, it is feasible to be more selective about acceptance criteria for donors of tissues containing living cells.

The Food and Drug Administration (FDA) and AATB require all donors of human tissues to be screened and tested for relevant communicable disease agents or diseases (RCDADs) as part of determining donor eligibility.^12^, ^13^, ^14^ This represents the first line of a multilayered approach to avoid communicable disease transmission via human tissues for transplantation. Tissue donor screening includes evaluating the donor's personal and social history, physical assessment/ examination, and medical records.^7^ Compared to the clinical setting, knowledge gaps exist regarding deceased donors,^15^ where information is obtained from next‐of‐kin, medical records, and available history.

The first known reported case of tissue transmission of MTB was via bone allografts reported by James et al. in 1953.^16^ The report indicated that four individuals who received bone allograft ribs from thoracoplasties performed in donors with pulmonary TB that had been frozen between −15 and −20°C in an antibiotic solution developed TB at the surgical implantation site. The practice of obtaining bone from TB‐ infected donors was subsequently abandoned as it became evident that the processing methods used did not prevent TB transmission.^16^

Bone allograft MTB transmission was next reported in 2021,^1^, ^17^ impacting 113 recipients (136 product units). The investigation revealed incomplete tissue recipient tracking and tracing^18^ and secondary transmission to healthcare workers.^19^ The donor was an 80 years old critically ill male, with a history of past residency and frequent travel to a country with TB incidence > 20/100,000 population. The donor exhibited signs and symptoms of TB infection during hospitalization that were attributed to other medical conditions. Seven recipients died before the outbreak was identified, one died the day after exposure notification, and all 105 remaining living recipients were treated for TB. The most recent reported TB transmission occurred in 2023 involving a US‐born individual, with no documented TB risk factors, with chest radiographs demonstrating infiltrates and a right upper lobe nodule, who died of pneumonia and sepsis. This transmission event was identified sooner after product distribution than in 2021, with two recipients dying among 34 total recipients. For the Centers for Disease Control and Prevention (CDC)‐investigated 2021 and 2023 cases, bone matrix allograft was confirmed to be the source of the transmission by genetic sequencing confirming the genetic relatedness of MTB isolates from donor products and recipients. Other TB tissue‐transmissions via medical products of human origin (MPHO) have occurred (Table 1).

**TABLE 1:** Reported *Mycobacteria tuberculosis* (MTB) transmissions via medical products of human origin (MPHO).

Tissue	Report dates	Notable findings
Heart valves^20^, ^21^	1972–1979^20^	‐ One case of miliary tuberculosis (TB) 8 months after valve replacement^20^
	1969–1974^21^	‐ Seven cases of miliary tuberculosis after valve replacement^21^ ‐ Diagnosed a few weeks to a year after surgery, all seven patients died ‐ All valves were from donors 60 and older
	‐ Streptomycin was added to the antibiotics mixture after these cases^20^ ‐ Many banks began culturing valves for TB, up until the 2000s, however, a survey revealed that due to consistent negative results, this practice was generally abandoned.^22^	
Bone (ribs)^16^	1953	‐ Ribs removed (often during thoracostomy for pulmonary tuberculosis) and stored frozen (−20 to −15°C) for use in orthopedic procedures^16^ ‐ Four cases of tuberculous abscesses following orthopedic spinal surgeries
Bone and bone marrow combination^1^, ^23^	2021	‐ 2021^1^ case: 113 recipients/eight deaths
	2023	‐ 2023^23^ case: 36 recipients/two deaths
Organ and hematopoietic stem cells (HSCs)	Multiple	TB transmission via organs^24^, ^25^, ^26^, ^27^ and HSC is well described,^28^, ^29^ with the incidence of TB in HSC recipients being about ten times less than that of solid organ transplant recipients.^28^

Table 1 provides information about reported cases of MTB transmission via MPHO documented in the literature. No reports exist of TB transmission via cornea transplantation, although there are indications it is feasible.^30^ In 1981, more strict donor eligibility criteria were implemented, and TB transmission declined and was not reported as a tissue‐transmitted infection since 1981 up to the recent events in 2021 and 2023. In reported cases of tissue‐transmitted MTB before 2020, allografts had been treated with antibiotics and/or other chemicals,^16^, ^20^, ^21^ and in recent cases, tissue was processed in a manner specifically intended to retain viable cells.^1^, ^23^

Tuberculosis is particularly challenging to evaluate in tissue donors for many reasons. TB infection has been found in most tissues and organs, is facile at evading the immune system, and organisms can remain dormant but viable in the body for decades awaiting an opportunity to reactivate when host immune function diminishes.^31^, ^32^, ^33^ Up to 13 million individuals in the United States are living with untreated, often undiagnosed, TB infection (TBI, e.g., latent TB infection (LTBI) and latent TB), and about 80% of the US TB cases result from reactivation of longstanding TBI.^34^ Higher rates exist in other geographic regions (see below). TBI is asymptomatic, tests used to detect MTB presence in living persons are imperfectly sensitive (and do not work in and are not approved for use in deceased individuals), and TB symptoms overlap many clinical syndromes. Clinical suspicion of TB (e.g., TB disease, active TB) is necessary to make a diagnosis because routine blood, sputum, or other cultures will not detect MTB—special test procedures must be ordered and performed, cultures may take up to 8 weeks to detect the organism, and culture‐negative pulmonary TB is probably under‐recognized.^35^ If TBI or TB remains undiagnosed prior to death, it is not feasible to make the diagnosis of TB solely by reviewing donor medical records—emphasizing the need for more stringent donor screening practices to elucidate risk factors for TB exposure and reactivation. It is essential to have heightened inclusion standards for donors of tissues containing viable cells and to ensure that donors meeting FDA sepsis criteria are excluded from donating tissues for transplantation.

## 2 EPIDEMIOLOGY AND CLINICAL OVERVIEW

### 2.1 Epidemiology

TB remains a global health concern as one of the leading infectious causes of death worldwide. In 2022, an estimated 10.6 million people worldwide had TB, killing about 1.3 million people.^36^, ^37^ According to CDC data, TB incidence has been declining in the United States since the 1950s.^38^ In 2022, the United States reported 8331 active TB cases, with an incidence of 2.5 cases per 100,000 persons, and about 602 TB deaths were reported in 2021.^39^, ^40^ Furthermore, following the coronavirus disease 2019 pandemic and for the first time in more than a decade, TB mortality has increased, and some models indicate that over the next 5 years, TB deaths in high‐burden settings could increase by up to 20% (Table S1).^37^, ^41^, ^42^

### 2.2 Transmission and pathogenesis

Most MTB transmissions occur from person to person via aerosolized particles. Both the innate and adaptive immune responses are crucial to the control of infection.^32^ In primary infection via the respiratory route, once aerosolized the bacteria are engulfed by the regional pulmonary macrophages and dendritic cells. During the primary phase, initial lung infection may occur, and viable bacteria spread to the regional lymph nodes with inflammation and fibrosis, creating the Ghon's complex. The inflammatory cells aggregate to form caseating granulomas, the pathological hallmark of TB.^43^ During primary infection, lymphohematogenous spread of MTB results in dissemination and seeding of distant organs, where various cell types can be infected besides phagocytes and dendritic cells, including endothelial cells, epithelial cells, fibroblasts and bone marrow stem and mesenchymal cells.^32^, ^44^, ^45^ During the latter stages of the primary infection, dendritic cells present bacterial antigens to T‐ lymphocytes activating the adaptive immune response, leading to the production of proinflammatory cytokines such as tumor necrosis factor, interleukin (IL)‐1B, IL‐12, and nitric oxide which augment the immune response to infection.

About 25% of infected individuals will have asymptomatic, latent infection with viable bacteria. About 5–10% of this group will develop reactivation or symptomatic disease, based on individual risk factors. Susceptibility to active TB is influenced by environmental, host, and pathogen factors.^46^ Mycobacteria evolved to express several mechanisms to evade the host immune system, allowing existence in dormant/ latent form in cellular phagosomes. MTB can reactivate and exit the intracellular environment and is transmissible to new hosts, most commonly via the respiratory route.^47^

### 2.3 Clinical and radiological manifestations

While MTB infection is often considered to be either “latent” (asymptomatic, i.e., TBI) or “active” (symptomatic and transmissible to others, TB, TB disease), it is more accurate to think of TB as a disease continuum. TB symptoms can follow primary infection or can appear years later as a reactivated disease. Symptoms onset is insidious over weeks and sometimes months. TB reactivates most commonly within the lungs (pulmonary TB), however approximately 19% of active TB cases in the United States are extrapulmonary (EPTB) (Table S2), 6% of cases have concurrent pulmonary and EPTB, and 2% have disseminated disease.^48^

There is considerable overlap between TB signs, symptoms, and radiological findings with those of other common chronic conditions such as heart, kidney, and liver failure, chronic obstructive pulmonary disease (COPD), and rheumatological and autoimmune disorders (Table S3). Given the overall lower incidence of TB in the United States, symptoms are more likely to be attributed to these common conditions, delaying diagnosis. As food and nutrition have become more accessible to people living in the United States, patients with TB may not appear thin or emaciated, but rather with normal weight or even obese, thus not fitting the classic picture of “consumption” described in the literature. Lastly, antibiotic overuse and inappropriate sample collection may result in negative smears and cultures, further hindering the diagnosis.

## 3 LITERATURE REVIEW

The WG reviewed available literature to formulate recommendations regarding donor screening practices for identifying donor risk factors for MTB. There are two categories upon which to assess risk; the risk of a donor having had exposure to MTB and the risk of reactivating TBI to active TB. The WG noted that some risk factors that are clinically important to consider, such as HIV disease, already exclude donors and are not considered in this review. Some risk factors reviewed were determined not evaluable in tissue donors, while others were determined to be exclusionary criteria for donors of either all tissues or of tissues containing viable cells. The WG is unaware of research specifically assessing the risk for the presence or transmission of MTB in donated tissues.

### 3.1 Exposure risk factors reviewed

MTB transmission occurs primarily by respiratory route from an individual with TB. The higher the incidence of TB in a particular setting and the greater the number of exposures, the higher the chance of MTB exposure and acquisition. In addition to countries with a higher TB incidence than the United States, various congregate settings were reviewed. The WG weighed factors including the availability and granularity of reliable data from public health authorities and donors/donor families, the likely magnitude of that risk, and the potential impact on donor loss. In many settings, there are scant or conflicting data, and units of measure are often not directly comparable. Most available data involve incident TB (i.e., newly diagnosed TB); TBI is more challenging to identify and less frequently assessed. Unless otherwise noted, TB incidence is expressed as a rate per 100,000 population.

#### 3.1.1 Geographic exposures

##### 3.1.1.1 Birth and residence

The United States has a low TB incidence, reported as 2.5 in 2022.^38^, ^40^ The US TB incidence rate is 17.1 times higher among non‐US‐born persons compared with US‐born persons, and 73% of TB cases occurred among non‐US‐born persons.^49^ Data indicate the risk of TB diagnosis is highest within the first two years of immigration (Table S4).

In 2022, 87% of the world's TB cases occurred in 30 high‐TB‐burden countries.^37^, ^50^ The World Health Organization (WHO) develops an annual TB global report^39^ providing incidence rates by country. These data are cited by the CDC, are comprehensive, regularly updated, and easily accessible; WHO data are expected to remain continually available. Though WHO‐estimated country incidence is a useful tool, it is not without limitations for donor screening purposes (Table S5).

In determining the incidence of concern for screening purposes, the WG aimed for consistency with current CDC screening guidelines. For determining patient TB screening guidelines, high‐burden countries are currently defined as those with an incidence of >20 per 100,000 individuals.^51^ For CDC international applicant screening (e.g., adoptees, immigrants, and refugees), several algorithms reviewed indicate frequent use of TB incidence ≥20 as a threshold.^52^ Of note, recent Immigration and Customs Enforcement (ICE) detainees may be of higher risk due to the combined risk of residence in a higher burden country and dwelling in a congregate setting during detention. A 2014–2016 study of ICE detention centers^53^ found a pulmonary TB incidence rate of 92.8/100,000 and 79.2% of those were asymptomatic at diagnosis. Even among patients with TB disease and positive AFB smears, 51.6 were asymptomatic, highlighting the importance of risk factor screening even in the absence of clinical symptoms.

##### 3.1.1.2 Travel

Travel to high‐incidence countries as a risk for TB is more difficult to characterize than country of birth. US surveillance does not capture travel‐related TB cases.^54^ However, many studies have demonstrated an increased risk for TB amongst long‐term travelers (Table S6).

The US Preventive Services Task Force recommends TB screening amongst those “who were born in or former residents of countries with increased Tuberculosis prevalence” but does not define the amount of residence in the country. A sample CDC TB risk assessment tool^34^ suggests screening for temporary or permanent residence of ≥1 month in a country with a high TB incidence. CDC core curriculum also describes increased risk individuals being those who “were born in or who frequently travel to countries where TB disease is common.”^55^ Another recent clinical recommendation is for “Persons who were born, lived, or had prolonged travel (>1 month) in these higher‐incidence settings should undergo testing for TBI…”.^33^ While various clinical TB risk‐factor screening criteria use either 1 or 3 months as a lower threshold for cumulative travel length, the only published data available at the time of this review supported an increased risk at 3 months or longer.^56^

#### 3.1.2 Homelessness

In comparison to the general population, persons experiencing homelessness (PEH) have an increased risk of acquiring MTB, harboring TBI, and progressing to active TB.^57^ 2021 data indicates PEH comprises 5% of US TB incidence.^58^, ^59^ Previously, annual TB incidence associated with homelessness was approximately 10‐fold^60^ to 20‐fold^61^ the rate in the general population. In 2006–2010, the estimated incidence was 36–47/100,000 homeless persons, in contrast to the overall TB incidence of 2.5–3.6.^60^

TB outbreaks/clusters among homeless persons in US cities have been associated with transmission at homeless shelters, single‐room occupancy hotels, rooming houses, prisons, and bars.^62^ In one study approximately 75% of persons with TB had lived in hotels within the past 2 years prior to TB diagnosis.^63^ A correlation was found between the length of time residing in a homeless shelter and rates of TB infection.^64^

In addition to the high incidence of TB incidence in PEH, TB outbreaks among PEH are associated with increased MTB transmission, often resulting in larger outbreak clusters.^60^ In one study, homeless persons with TB were often highly contagious at the time of diagnosis as demonstrated by the large proportion of patients who had cavitating pulmonary disease and sputum smears with numerous acid‐fast bacilli.^62^ As shown in these studies, poverty and crowded and close living conditions associated with PEH create an opportune environment for TB transmission.^65^ Limitations of detection include the transient nature of PEH,^65^ overcrowded living, limited testing, and the reluctance of PEH to provide accurate health history.

#### 3.1.3 Correctional facilities

##### 3.1.3.1 Incarceration

The global incidence rate of TB among incarcerated people (IP) is estimated to be 1148 but varies greatly by WHO region, and in North America, this rate is less than 50.^66^, ^67^, ^68^, ^69^ In the United States, 2.4% of incident TB is among IP^58^, ^59^ and TB risk in IP varies by setting. A US study by the CDC TB elimination group found the TB incidence in US IP to range from 8 to 29 depending on the type of prison, with local jails having the highest incidence rates compared to other types of prisons.^58^, ^70^, ^71^ Most studies report an incidence twice that of the general population with some states reaching incidence rates comparable to intermediate‐TB‐burden regions.^72^

##### 3.1.3.2 Correctional facility workers

The few studies of TB rates in correctional facility workers (CFW) suggest that incidence among CFW is increased but closer to the general population than to IP. Systematic review and meta‐analysis evaluating latent and active TB in CFW in six countries between 1987 and 2017^68^ demonstrated a pooled latent TB incidence of 2%. US CFW TBI incidence varied depending on the year and the studies, with an incidence of 1%–6% in the 1990s to an incidence of 0.4% in 2010.

Purified protein derivative tests were performed in adult CF health workers (high‐exposure risk) in four US CFs. Results showed an overall TB skin test prevalence rate of 17.7%, reactivity rate of 2.2%, and annual incidence of 1.3%. In multivariate analysis after controlling for Bacillus Calmette‐Guérin vaccination, only origin of birth remained significantly associated with TB prevalence among study participants, indicating that the risk factors were predominantly demographic rather than occupational.^73^

#### 3.1.4 Hospitals and general healthcare facilities

CDC surveillance data (1995–2007) demonstrated TB incidence rates among health care workers (HCWs) like those in the general population.^74^, ^75^ A retrospective HCW cohort study (1998–2014) in a low‐TB‐incidence state found a 0.3% TB skin test conversion rate, with a limited proportion due to occupational exposure; based on a literature review, it appeared that the risk of HCW contracting TB was decreasing.^76^ In 2015 a National TB Controllers Association‐CDC work group reviewed 1147 citations, of which 39 studies involved HCW among whom a low proportion test positive and converted during serial testing.^74^ High‐risk HCW exposures are summarized (Table S7).

#### 3.1.5 Long‐term care facilities

##### 3.1.5.1 Residents

Almost 25% of all US TB cases are in those age 65 or older, who also comprise most individuals living in nursing homes (NH) or other long‐term care facilities (LTC)—about 1.5% of 2021 US TB incidence.^58^, ^59^ Published data regarding rates of TBI or TB in LTC are sparse. In July 1990, the CDC reported a 1984–1985 NH TB incidence of 39.2.^77^ Causes identified include past MTB exposure, immune senescence, immunosuppressive agents, co‐morbidities, malnutrition, and close contact with other residents (i.e., congregate setting).^78^ Accordingly, the CDC recommended^79^ TB testing of LTC residents upon entry. A study of California NH residents (2000–2009) indicates substantial improvements in TB rates. Notably, TB incidence among NH residents improved more than among community‐dwelling adults and with a lower overall incidence—likely due to intensive public health interventions.^80^

##### Employees

NH employees historically had increased TB risk. Prior to implementing TB screening and surveillance, the reported incidence (1990) was three times higher than other employees.^79^ Recent data on NH employees are not available, but the improvements in rates of TB amongst NH residents and HCWs were previously noted.

#### 3.1.6 Reactivation risk factors

A well‐functioning immune system is necessary to prevent TBI/ activation. The WG reviewed multiple risk factors that increase the risk for reactivation. Some conditions can increase both the likelihood of MTB exposure and reactivation disease, and the WG classified such risk factors into categories most contributory to risk.

#### 3.1.7 Chronic kidney disease

Chronic kidney disease (CKD) is a growing global health issue with an estimated 2017 prevalence of 9.1% worldwide^81^ with prevalence increasing in TB endemic regions.^82^ There is elevated TB risk in cohorts of people with CKD.^83^, ^84^, ^85^, ^86^ Multiple factors lead to immune dysfunction in CKD with the associated increased risk of infections^86^, ^87^, ^88^, ^89^ including TB (Table S8).

CKD has a multi‐factorial effect on an individual's immune status due to cellular immunity changes from low albumin, uremia, vitamin D deficiency, and malnutrition,^90^ in addition to the additive effects from any comorbidities occurring with CKD, such as diabetes. CKD often requires frequent medical visits, which increases the likelihood of nosocomial exposures.^87^

Though an increased risk for TB disease is observed in CKD, the relative and hazard risk range of 1.2–2.9 in recent studies (Table S9) is not supportive of CKD overall (i.e., all stages 1–5 when data are combined) as a strong enough independent risk factor to preclude donation of viable cell tissues. This finding reflects epidemiologic studies adjusting for confounders such as diabetes, HIV, immunosuppressive drugs, lifestyle, and socioeconomic factors. Many of these factors have already been accounted for in our exposure and reactivation risk stratification.

However, advanced renal disease (CKD Stage 4–5) prior to dialysis initiation or transplant is of somewhat higher risk than earlier stages 1–3 (incident rate ratio of 3.6 combined).^91^

#### 3.1.8 End‐stage renal disease and dialysis

Immune dysfunction in end‐stage renal disease, including individuals on dialysis, is well‐described and understood^92^, ^93^, ^94^; multiple studies over decades indicate that individuals on dialysis have a higher incidence of TB, while the degree and assessment of increased risk differed over time and location (Table S10). Though some outbreaks of TB in dialysis centers are reported,^95^, ^96^ the last reported outbreak in a US dialysis center was in 2004,^96^ likely reflecting public health efforts to mitigate TB and other infectious disease risks in this population.^97^, ^98^

#### 3.1.9 Diabetes

The interaction between TB and diabetes mellitus (DM) is long‐recognized, complex, and difficult to quantify. In 2021, DM was the most commonly reported TB risk factor, reported in 23.9% of incident TB.^58^ Like CKD, TB and DM are considered “syndemic”, in that these two highly prevalent diseases frequently co‐occur^37^, ^99^, ^100^ and negatively impact the frequency and severity of each other.^99^, ^101^, ^102^, ^103^ The risk of developing TB is two to three times higher amongst diabetics,^84^, ^103^, ^104^, ^105^, ^106^, ^107^, ^108^, ^109^, ^110^ diabetes management is more difficult in the presence of TB,^111^ the severity of TB is worse with diabetes,^102^, ^108^ and DM impairs immune function^110^, ^112^ negatively impacting the response to TB treatment.^101^, ^103^, ^104^, ^105^, ^112^, ^113^, ^114^, ^115^ Glycemic control (in DM or pre‐diabetes) impacts TB but data are mixed without clear criteria^103^, ^104^, ^108^, ^110^, ^116^, ^117^; evaluating the level of glycemic control is impracticable as a donor screening tool.

#### 3.1.10 Cirrhosis

Cirrhosis is associated with immune dysfunction, leading to abnormalities in both innate and adaptive immunity and immune paralysis.^118^, ^119^, ^120^, ^121^, ^122^ TB and cirrhosis of the liver are endemic in many regions of the world.^123^

A large national longitudinal study from Taiwan (>40,000 cirrhosis and >200,000 non‐cirrhosis) concluded that cirrhotic patients are at a greater risk of TB compared with non‐cirrhotic patients (*p* < 0.001) adjusted for underlying conditions and associated factors. Among cirrhotic individuals, males, diabetes, chronic renal failure, COPD, and silicosis were associated with higher rates of TB infection; in contrast, cirrhosis was associated with autoimmune disease and cancer was not associated with higher TB disease. Among etiological bases of cirrhosis, alcoholism, and hepatitis C infection were associated with significantly higher TB risk levels.^124^ A Danish population‐based study had similar findings, where the cirrhotic/noncirrhotic TB incidence rate ratio in the general population, age 55–64 years, was 27.0 and 34.3 in women.^125^

Cirrhosis has more frequent EPTB involvement, including peritoneal and miliary TB.^126^, ^127^ Moreover, in cirrhosis TB constitutes a diagnostic dilemma, diagnosis is often missed or delayed because of non‐specific findings and overlap with symptoms of chronic liver disease.^128^

#### 3.1.11 Alcohol use disorder and alcoholic liver disease

Alcohol use disorder is one of the most common global risk factors for TB.^37^, ^129^ Alcohol consumption impairs immune function and alveolar monocyte‐ macrophage function, increasing susceptibility to acquiring TB infection as well as reactivating latent infection.^119^, ^129^, ^130^, ^131^ In mice, alcohol inhibits granuloma formation, IL‐2 production, interferon‐gamma production, and CD4^+^ proliferation.^132^ Alcohol consumption is further associated with liver disease, malnutrition, and social characteristics with an increased risk of spreading TB.^133^ In males (more than females), heavy use of alcohol over time was found to be associated with an increased risk of contracting TB and increased morbidity and mortality.^134^

A 2008 meta‐analysis of 21 publications^135^ reported low to moderate alcohol intake was not associated with an increased risk of TB. A 2009 review found 2.9 times increased TB risk among heavy drinkers.^136^ In 2021, the US reports excess alcohol use among 8.2% of persons with TB age ≥ 15.^58^, ^59^

#### 3.1.12 Organ transplantation

Solid organ transplant (SOT) recipients have varied degrees of immune suppression, placing them at a higher risk for infections than the general population.^137^, ^138^ TB incidence among SOT recipients in 2005 was estimated to be 20–74 times higher than that for the general population, with high mortality.^139^ The risk of SOT recipients developing TB varies by geographic location, age, type of organ received, and type of immunosuppression.

A study conducted in a large university medical center in New York found in post‐SOT recipients found a cumulative TB incidence of 264, and was higher in minorities and kidney transplant recipients.^140^ Other studies showed similar increases.^141^ A large Spanish prospective study found TB incidence was increased among SOT recipients; risk factors identified were older age and receipt of a lung transplant and TB‐attributable mortality was 9.5%.^142^ Another review, among all known post‐SOT TB cases between 1998 and 2016, found that disseminated and EPTB occurred in 15.96% and 29.84%, respectively.^143^ Careful recipient screening and donor selection may not prevent the development of TB after SOT; TB diagnosis in SOT recipients is often delayed because clinical manifestations may be muted and TB testing may be falsely negative or indeterminate.^144^

#### 3.1.13 HSC transplantation

Hematopoietic stem cell transplantation (HSCT) is associated with some TB‐associated risks. TB disease after HSC is less frequent than after SOT (presumably due to longer immunosuppression in SOT),^28^ and reported to be 3–10^29^ (2021) or 10–40^28^ (2014) times more common in HSCT recipients than the general population. Graft versus host disease in allogeneic HSC recipients also adds to infectious risk including for MTB. Hematologic malignancies may be associated with immune dysfunction depending on the primary disorder; treatments typically include cytotoxic drugs, corticosteroids, irradiation, and other immunosuppressive therapies.^28^ Although exact data are unavailable, TB occurs more frequently in allogeneic than autologous HSCT recipients, likely due to donor‐derived T lymphocyte function remaining altered for ∼1 year after transplant.^145^

#### 3.1.14 Immunosuppressive medications

A principal risk factor for TB reactivation in those with TBI is immune suppression or immune incompetence, with the latter including primary immune‐deficiency diseases and secondary immunodeficiency (e.g., medication‐induced).^146^ Systemic immunosuppression is a risk factor for TBI reactivation and the development of TB. Immune changes in patients taking immunosuppressive medications (ISM) are multifactorial and related to ISM categories (Table S11). ISM may impact immunological pathways integral to controlling anti‐mycobacterial activity.^147^ A detailed description of the impact of various ISMs on MTB is provided (Table S12).

#### 3.1.15 Age ≥ 65

Increased age is associated with increased TB incidence and severity,^148^ representing both cumulative exposure and increased risk of reactivation due to immune senescence.^149^ In the United States, while TB overall incidence has been decreasing,^150^ the proportion of TB cases diagnosed in people aged ≥ 65 has been increasing.^150^ About 25% of new US cases are among people aged ≥ 65,^78^ with yearly rates about 1.5 times that of adults aged 21–64 (Table S13).^149^, ^151^ Atypical presentations in advanced age and the impact of immune senescence on TB testing are consistent with the missed TB infections in hospitalized patients in recent TB transmission cases.^1^, ^17^, ^23^

#### 3.1.16 Testing

Demonstrating TB infection relies on microbiologic detection in clinical samples from symptomatic individuals. TBI is asymptomatic, and no gold‐standard test exists for determining whether an individual has been exposed to MTB, complicating the ability to fully assess TBI testing accuracy in studies. Routine testing for TBI (i.e., without known risk factors for exposure) is not clinically recommended in low‐incidence settings like the United States,^33^, ^152^ and there is insufficient evidence to support it in the organ donation setting.^26^

Clinical testing to determine MTB presence is accomplished by TB skin testing (TST) or interferon‐gamma release assays (IGRA)—neither of which can discern between TBI and TB disease.^26^, ^33^, ^153^, ^154^, ^155^, ^156^ TST cannot be performed in deceased donors, and because documentation of results requires time to elapse and a return visit, it is not practicable for living tissue donors. IGRA assays are based on the exposure of T‐cells in a blood specimen to TB antigens, measuring T‐cell activation by measuring cytokine release.^26^, ^33^, ^148^, ^153^ IGRA sensitivity in non‐deceased populations ranges from 70% to 90%,^33^, ^153^, ^156^ and specificity estimates in some high‐exposure‐risk populations are estimated up to >90%.^33^ There is inadequate data available regarding IGRA assay performance in organ donors,^26^, ^148^ clinical issues (including steroid administration or sepsis) can negatively impact results, and IGRA testing has not been evaluated in deceased donors. Importantly, IGRA requires living cells, with resulting infeasible specimen and logistical requirements (Table S14) for deceased tissue donors. Testing donors lacking exposure risk factors increases the likelihood of false positive results; negative IGRA results cannot adequately rule out TB infection.^26^, ^33^, ^156^, ^157^ Furthermore, IGRA tends to yield a high percentage of indeterminate/ invalid results, particularly in critically ill or otherwise immune‐suppressed individuals,^33^, ^153^, ^156^, ^158^, ^159^, ^160^, ^161^ who are at increased risk of TB reactivation. Requiring a negative IGRA result, which cannot rule out TBI or TB, to permit tissue donation could result in unacceptable donor loss for questionable return and alarmingly, could provide a false sense of security when donors test negative. For these reasons, MTB WG recommends against testing deceased tissue donors using IGRA or TST.

Product‐specific TB testing is an option but must be approached with extreme caution. It is notable that in the second TB transmission episode, polymerase chain reaction testing was performed but failed to detect MTB.^23^ MTB is intracellular, slow growing, requires special culture media, and EPTB is often paucibacillary (tends to have a small number of organisms present).^162^ Culture is the standard for laboratory detection of MTB, having the highest sensitivity and specificity.^154^ Positive results may take 4–6 weeks, negative results require 8 weeks. Although nucleic acid amplification testing (NAT) for MTB may have comparable sensitivity to culture only in a liquid sample matrix, tissue products are not liquid. Cultures would be necessary in addition to NAT testing. Whether testing by culture or NAT, samples must undergo preparation to release the (intracellular) MTB to permit detection without also killing or breaking up MTB bacilli (preventing detection). Tissue sampling for testing is a major challenge. For example, in animal disease surveillance or human clinical settings, lesions are tested to improve the detection sensitivity, but in tissue donation tissues with visible abnormal lesions are avoided and not procured. In EPTB, few organisms are generally present, and samples must contain adequate numbers of organisms to permit microbial detection. Since it is not generally feasible to create a homogenous mixture of tissue, an adequate sample size (which must be validated) may be large. Because of the sampling challenges associated with MTB in tissues, positive results would exclude the use of any tissues from that donor, but, importantly, negative results cannot definitively preclude the presence of MTB in donated tissues. While the TB culture of the product is appropriate in the tissue setting, it cannot safely replace careful donor selection. If the culture of tissue is desired, each individual tissue product type from each tissue bank and each sampling and testing methodology would have to undergo extensive validation studies.

### 3.2 Clinical issues to consider

#### 3.2.1 Sepsis syndrome of undetermined etiologic agent

The Third International Consensus Definitions for Sepsis and Septic Shock (Sepsis‐3) defined sepsis as “a life‐threatening organ dysfunction caused by a dysregulated host response to infection.”^163^ The Health Care Cost & Utilization Project (HCUP) found that of the 10 most common principal diagnoses in 2018, septicemia was the most frequent, making a sepsis diagnosis one of the most encountered diagnoses during donor selection as well.^164^ In sepsis, a causative organism is frequently not identified (in up to 50 percent of patients), the proportion of culture‐negative sepsis^165^ is rising, is associated with greater acute organ dysfunction and mortality, and is an independent predictor of death.^165^ Differentiating true culture‐negative sepsis from systemic inflammatory responses due to non‐infectious conditions is challenging, especially postmortem. In donor selection, the WG recommends against accepting donations from donors diagnosed with sepsis at the time of death. MTB is not yet an‐FDA identified RCDAD, but severely ill deceased donors meeting FDA donor screening criteria for sepsis^13^ are required to be excluded from donation. Even before recent transmissions, AATB standards required that individuals identified with active infections, explicitly including TB, be excluded from donation.^14^ Careful chart review is essential to exclude donors meeting sepsis criteria.

#### 3.2.2 Chronic illness and over all poor health

Chronically ill Individuals, especially those with multiple chronic conditions, are at risk of poor overall health and frailty regardless of age. Those patients are at increased risk of significant adverse health outcomes, including infections,^166^, ^167^ highlighted by the Advisory Committee on Immunization Practices recommendations for additional vaccination against infections in this population.^168^ Differentiating the effects of an occult infection from systemic symptoms due to other non‐infectious conditions in chronically ill and frail individuals, regardless of age, can be challenging, especially postmortem. In donor selection, the WG advises extreme caution in accepting viable cell donation under these circumstances.

## 4 AATB TISSUE DONOR SCREENING REQUIREMENTS

The AATB Physicians Council MTB WG met approximately every other week between November 2022 and March 2024. TB experts, including from the federal government, presented to the WG and provided useful references. Available clinical literature was reviewed, expert opinion, including considerations of balancing risk with tissue availability, was applied, and consensus was reached through discussion to determine final donor evaluation criteria. Commentary on each of the donor screening criteria is provided in Table S15. The donor screening criteria recommended by the WG are provided in Table 2.

**TABLE 2:** Tuberculosis (TB) risk factor tissue donor exclusion criteria.

Potential donors with the following are ineligible to donate *tissues* Persons with a history (ever) of tuberculosis disease (sometimes referred to as “active” or “clinically active” tuberculosis) Persons with a history of (latent) tuberculosis infection initially diagnosed within the past two (2) years (i.e., the individual has had a positive test for tuberculosis)^†^	
For tissues intended to ultimately retain viable cells, (i.e., all products comprising or containing tissues that are processed in a manner to retain living cells), the following are ineligible to donate, including persons: aged ≥ 65 who, within the past 2 years, traveled for ≥3 months or immigrated from a country with the most current available tuberculosis incidence of ≥ 20 (rate per 100,000 population) available on the WHO TB country profile website: https://worldhealthorg.shinyapps.io/tb_profiles/ with exposure to an individual with tuberculosis disease within the past 2 years with (latent) tuberculosis infection > 2 years ago (i.e., positive TB test > 2 years ago) experiencing homelessness housed in shelters or other congregate settings, ≤2 years ago have been incarcerated ≤2 years ago with ESRD (CKD 5) with or without dialysis who have received a solid organ transplant	
Potential donors **with at least one risk factor from each column below for exposure and reactivation** are *ineligible to donate* ***tissues intended to ultimately retain viable cells*,** including persons:	

**Exposure risk factors**	**Reactivation risk factors**
who had birth, travel, or residence ≥3 months cumulative in a country with the most current available tuberculosis incidence of ≥ 20 (rate per 100,000 population) that occurred >2 years ago,	with advanced kidney disease, pre‐dialysis—otherwise known as CKD Stage 4, GFR < 30
ever experiencing homelessness and were housed in shelters or other congregate settings > 2 years ago	with diabetes mellitus
have been incarcerated > 2 years ago	with cirrhosis or alcoholic liver disease
who have had exposure to an individual with tuberculosis disease > 2 years ago	with alcohol use disorder/ excessive or heavy alcohol use
	who use immunosuppressive medications

^†^ Tests for tuberculosis include TB skin test (TST; other names used interchangeably—purified protein derivative [PPD] or Mantoux) and Interferon Gamma Release Assay (IGRA) blood tests (e.g., QuantiFERON‐TB Gold, T‐SPOT).

Table 2 provides a listing of all TB‐related tissue donor exclusionary criteria recommended by AATB.

## 5 CONCLUSIONS

These donor screening criteria represent minimal criteria for exclusion and do not exclude all possible donor TB risks. TB is particularly challenging because it can be asymptomatic or mimic other, more common diseases. Careful donor evaluation is critical to tissue safety and is part of overlapping layers of safety.^169^, ^170^ While donor testing would be a welcomed additional layer of safety, this is not currently feasible; research leading to test development is urged.

Tissue transplantation has an overall excellent safety record, but diligence is warranted. As tissues containing viable cells have become more frequently used, the need for stricter donor acceptance criteria for these tissue products is clear. As with any substance of human origin, it is not possible to fully eliminate the communicable disease risk to recipients. Lessons learned include the need for meticulous reviews of available clinical data, clinician education about human tissues and communicable disease risks, informed consent of tissue recipients, improved tracking and tracing of tissue products, and the need for recipient follow‐up.

## Supporting information

Supporting Information

Supporting Information

Supporting Information

Supporting Information

Supporting Information

Supporting Information

Supporting Information

Supporting Information

Supporting Information

Supporting Information

Supporting Information

Supporting Information

Supporting Information

Supporting Information

Supporting Information

## Data Availability

Data sharing is not applicable to this article as no new data were created or analyzed in this study.
