# Supplementary material for: The American Association of Tissue Banks tissue donor screening for Mycobacterium tuberculosis—Recommended criteria and literature review
Source: Transpl Infect Dis. 2024 Jun 9;26(Suppl 1):e14294. doi: 10.1111/tid.14294 (PMC11578281; doi:10.1111/tid.14294)
Supplement: Supplementary file 1 — Supporting Information [file TID-26-e14294-s007.docx]

**Supp Table 1. Coronavirus 2019 (COVID-19) impact on Tuberculosis (TB)**

| **Incidence of TB during COVID-19 pandemic** | **TB Detection, Reporting &Treatment during COVID-19 pandemic** | **Long term effects of COVID-19 pandemic on TB** |
| --- | --- | --- |
| Globally and in the United States (US), there was a substantial fall  (-18%) in the number of people newly diagnosed with TB and reported to national authorities in 2020 compared to 2019 and in marked contrast to global reporting increases between 2017 and 2019.  The pattern reversed in 2021.^1,2^ | Available data suggest that there were delays in TB diagnosis, either due to the widespread disruptions to healthcare or missed diagnosis of TB symptoms given the similarities between COVID-19 and TB disease.  CDC case reports showed that some people with TB disease were evaluated for COVID-19, but not tested for TB, during multiple encounters with healthcare systems.^2^  It is also possible that person to person MTB transmission was reduced during the pandemic, due to social distancing, mask wearing, and limited mobility.^3,4^ | Following the COVID-19 pandemic and for the first time in more than a decade, TB mortality has increased.  Some models indicate that over the next 5 years TB deaths in high-burden settings could increase by up to 20%.^1,3,5^ |

**Supp Table 1** provides an overview of the impact of the Coronavirus 2019 (COVID-19) pandemic on tuberculosis (TB). The impact of the COVID 19 pandemic on TB is hard to understand fully but available data indicates that the pandemic adversely affected detection, reporting, and treatment of TB.

References:

1. World Health Organization (WHO). *Global Tuberculosis Report 2022*.; 2022. Accessed January 22, 2024. https://www.who.int/teams/global-tuberculosis-programme/tb-reports/global-tuberculosis-report-2022

2. Centers for Disease Control and Prevention. Effect of COVID-19 on Tuberculosis in the U.S. CDC Newsroom Releases. Published March 24, 2022. Accessed January 29, 2024. https://www.cdc.gov/media/releases/2022/s0324-tuberculosis-covid-19.html#:~:text=Case%20reports%20have%20revealed%20some,more%20advanced%20stages%20of%20disease

3. Zimmer AJ, Klinton JS, Oga-Omenka C, et al. Tuberculosis in times of COVID-19. *J Epidemiol Community Health (1978)*. 2022;76(3):310-316. doi:10.1136/jech-2021-217529

4. Kyu HH, Ledesma JR. What is the impact of the COVID-19 pandemic on tuberculosis? *Lancet Glob Health*. 2023;11(9):e1323-e1324. doi:10.1016/S2214-109X(23)00360-1

5. Hogan AB, Jewell BL, Sherrard-Smith E, et al. Potential impact of the COVID-19 pandemic on HIV, tuberculosis, and malaria in low-income and middle-income countries: a modelling study. *Lancet Glob Health*. 2020;8(9):e1132-e1141. doi:10.1016/S2214-109X(20)30288-6
