# Supplementary material for: The American Association of Tissue Banks tissue donor screening for Mycobacterium tuberculosis—Recommended criteria and literature review
Source: Transpl Infect Dis. 2024 Jun 9;26(Suppl 1):e14294. doi: 10.1111/tid.14294 (PMC11578281; doi:10.1111/tid.14294)
Supplement: Supplementary file 2 — Supporting Information [file TID-26-e14294-s003.docx]

**Supp Table 2. Extra-pulmonary Tuberculosis (EPTB): Reported Sites and Frequency**

| **Overall Extrapulmonary TB (EPTB) Disease in US in 2022**^1^   - TB cases with ONLY extrapulmonary disease represented 18.8% in 2022 per the CDC - Cases with BOTH pulmonary and extrapulmonary involvement were 10.6% of total cases | | |
| --- | --- | --- |
| **Site** | **Percentage (%) of Extrapulmonary Cases involving this site**^1^ | **Key Points** |
| Lymphadenitis ^2^ | 26.1 | - Infection of lymph nodes - Typically cervical |
| Pleural^2^ | 22.1 | - Often results in pleural effusions |
| Spine (“Pott’s disease”), bone,  joint ^3–6^ | 9.4 | - Large joints more commonly affected - Often from reactivation |
| Gastrointestinal/ Abdominal^7^ | Percentage not reported overall  (Peritoneal was 6.3) | - Ileocecal most often - Can result in peritonitis, ascites, ulcers, strictures |
| Central Nervous System^8^ | 5.8 (Only Meningeal reported) | - Meningitis, encephalitis - Abscess - Tuberculoma |
| Urogenital^9–12^ | 4.2 | - Males and female fertility can be affected |
| Cutaneous^13–18^ | (Percentage not reported) |  |
| Ocular^19–23^ | (Percentage not reported) |  |
| Disseminated/  Miliary ^24-24^ | Considered a form of pulmonary disease for CDC surveillance purposes, therefore not represented.  Cases with both pulmonary and extrapulmonary sites, however, were 10.6% ^1^ | - Systemic infection caused by widespread hematogenous spread - Predominantly affecting highly vascularized organs – (lungs, liver, spleen, bone marrow and kidneys.^24^ ) - Can be from early disease or reactivation - Highest risk = Infants, children< 5 years old, elderly and immunocompromised.^25^ |
| Other sites^1^ | Laryngeal 1.1^7^  24.5 (overall) |  |

**Supp Table 2** provides information about the sites of EPTB, reported frequency, and key points. EPTB is more difficult to diagnose, and is highest risk for tissue transmission.

References:

1. Centers for Disease Control and Prevention, Division of Tuberculosis Elimination. Reported Tuberculosis in the United States, 2022. Tuberculosis (TB) [CDC Website]. Published July 8, 2023. Accessed February 13, 2024. https://www.cdc.gov/tb/statistics/reports/2022/table19.htm

2. Peto HM, Pratt RH, Harrington TA, LoBue PA, Armstrong LR. Epidemiology of Extrapulmonary Tuberculosis in the United States, 1993–2006. *Clinical Infectious Diseases*. 2009;49(9):1350-1357. doi:10.1086/605559

3. Pigrau-Serrallach C, Rodríguez-Pardo D. Bone and joint tuberculosis. *European Spine Journal*. 2013;22(S4):556-566. doi:10.1007/s00586-012-2331-y

4. Khanna K, Sabharwal S. Spinal tuberculosis: a comprehensive review for the modern spine surgeon. *The Spine Journal*. 2019;19(11):1858-1870. doi:10.1016/j.spinee.2019.05.002

5. Gardam M, Lim S. Mycobacterial Osteomyelitis and Arthritis. *Infect Dis Clin North Am*. 2005;19(4):819-830. doi:10.1016/j.idc.2005.07.008

6. Dobson J. Percivall Pott. *Ann R Coll Surg Engl*. 1972;50(1):54-65.

7. Chakinala RC, Khatri AM. Gastrointestinal Tuberculosis. *StatPearls [Internet]*. Published online January 2024.

8. Rock RB, Olin M, Baker CA, Molitor TW, Peterson PK. Central Nervous System Tuberculosis: Pathogenesis and Clinical Aspects. *Clin Microbiol Rev*. 2008;21(2):243-261. doi:10.1128/CMR.00042-07

9. Ravikanth R, Kamalasekar K, Patel N. Extensive primary male genital tuberculosis. *J Hum Reprod Sci*. 2019;12(3):258. doi:10.4103/jhrs.JHRS_3_19

10. Muneer A, Macrae B, Krishnamoorthy S, Zumla A. Urogenital tuberculosis — epidemiology, pathogenesis and clinical features. *Nat Rev Urol*. 2019;16(10):573-598. doi:10.1038/s41585-019-0228-9

11. Malik S. Genital Tuberculosis and its Impact on Male and Female Infertility. *US Endocrinol*. 2020;16(2):97. doi:10.17925/USE.2020.16.2.97

12. Chatterjee S. Latent Genital Tuberculosis In Male - A Possible Cause Of Reproductive Failure. *Reproductive Medicine, Gynecology & Obstetrics*. 2020;5(4):1-6. doi:10.24966/RMGO-2574/100057

13. van Heerden JK, Broadhurst AGB, de Jager RS, et al. Cutaneous tuberculosis: An infrequent manifestation of a common pathogen in South Africa. *S Afr J Infect Dis*. 2023;38(1). doi:10.4102/sajid.v38i1.526

14. Ngan V, Oakley A, DermNet NZ. Cutaneous tuberculosis. DermNet. Published 2023. Accessed August 8, 2023. https://dermnetnz.org/topics/cutaneous-tuberculosis

15. Nguyen KH, Alcantara CA, Glassman I, et al. Cutaneous Manifestations of Mycobacterium tuberculosis: A Literature Review. *Pathogens*. 2023;12(7):920. doi:10.3390/pathogens12070920

16. Santos JB dos, Figueiredo AR, Ferraz CE, Oliveira MH de, Silva PG da, Medeiros VLS de. Cutaneous tuberculosis: epidemiologic, etiopathogenic and clinical aspects - Part I. *An Bras Dermatol*. 2014;89(2):219-228. doi:10.1590/abd1806-4841.20142334

17. Dias MFRG, Bernardes Filho F, Quaresma MV, Nascimento LV do, Nery JA da C, Azulay DR. Update on cutaneous tuberculosis. *An Bras Dermatol*. 2014;89(6):925-938. doi:10.1590/abd1806-4841.20142998

18. Yadav D, Khan JA. Undermined Skin Lesion Emerged as Tubercular Meningitis: A Case Report. *Journal of Nepal Medical Association*. 2023;61(259):280-282. doi:10.31729/jnma.8079

19. Albert DM, Raven ML. Ocular Tuberculosis. *Microbiol Spectr*. 2016;4(6). doi:10.1128/microbiolspec.TNMI7-0001-2016

20. Rathinam S. Tuberculosis and the Eye. UpToDate. Published 2023. Accessed August 7, 2023. https://www.uptodate.com/contents/tuberculosis-and-the-eye

21. O’Keefe GD, Pakravan M, Hossain HA. Ophthalmologic Findings Related to Tuberculosis. American Academy of Ophthalmology EyeWiki. Published December 31, 2023. Accessed August 7, 2023. https://eyewiki.aao.org/Ophthalmologic_Findings_Related_to_Tuberculosis

22. Neuhouser AJ, Sallam A. *Ocular Tuberculosis*.; 2024. Accessed August 7, 2023. https://www.ncbi.nlm.nih.gov/books/NBK559303/

23. Basu S. Absence of Evidence as The Evidence Of Absence: The Curious Case of Latent Infection Causing Ocular Tuberculosis. *Frontiers in Ophthalmology*. 2022;2. doi:10.3389/fopht.2022.874400

24. Sharma SK, Mohan A, Sharma A, Mitra DK. Miliary tuberculosis: new insights into an old disease. *Lancet Infect Dis*. 2005;5(7):415-430. doi:10.1016/S1473-3099(05)70163-8

25. Thwaites G. 40 - Tuberculosis. In: *Manson’s Tropical Infectious Diseases (Twenty-Third Edition)*. 23rd ed. ; 2014:468-505.e3.
