# Supplementary material for: The American Association of Tissue Banks tissue donor screening for Mycobacterium tuberculosis—Recommended criteria and literature review
Source: Transpl Infect Dis. 2024 Jun 9;26(Suppl 1):e14294. doi: 10.1111/tid.14294 (PMC11578281; doi:10.1111/tid.14294)
Supplement: Supplementary file 3 — Supporting Information [file TID-26-e14294-s010.docx]

**Supp Table 3. Pulmonary Tuberculosis (TB) Clinical & Radiological Manifestations**

| **Common TB Clinical Manifestations** | **Common TB Radiological Manifestations** |
| --- | --- |
| The most recognized symptom is persistent cough over 2 weeks, reported in 95 %  Fever, weight loss and night sweats occur in about 75%, 45% and 55 % of patients respectively.^1^  Sputum can be mucoid, purulent, blood-stained, or the patient may have frank hemoptysis.  On physical exam, lung auscultation findings may be subtle compared to radiological findings.^2^ | Radiological manifestations of pulmonary TB can be helpful for early recognition and diagnosis^3^ but the classical “evolution” of radiographic appearance of TB does not reliably occur, and the chest x-ray cannot reliably rule-in or rule-out TB.^4^  Pulmonary apical lesions represent the most common abnormality found in reactivated TB, may appear as consolidation, extensive pleural thickening and fibrosis, fibro-nodularity in a tree and bud or miliary pattern, a nodule and as cavitations.  Apical fibrosis with fibronodular changes, nodules and cavitations can result from other non-tuberculous but potentially transmissible infectious diseases that also can lie dormant until they reactivate and spread include aspergillosis,^5^ melioidosis,^6^ histoplasmosis,^7^ cryptococcosis, ^8^ and others.  In contrast, apical caps of isolated pleural thickening of less than 5 mm are more common, often associated with age,^9,10^ tall and thin body habitus,^10,11^ and are usually benign and are of no clinical significance.^9,12,13^  Prospective viable tissue donors with significant pulmonary apical changes, including pleural thickening (>5 mm) or scarring, and nodules interpreted as possible granulomata have an increased risk for TB and should be strongly considered for donor disqualification.  Other radiological findings of TB include intrathoracic lymphadenopathy and pleural effusion.  Findings in TB can be nonspecific and mimic other lung and heart conditions such as pleural effusion and diffuse opacities often interpreted as pulmonary edema, aspiration pneumonitis, ventilator associated pneumonia in hospitalized patients, leading to missed TB diagnosis.  Severely immune-suppressed patients and young children are less likely to present with cavitation on chest X-ray, and more frequently have bilateral and lower lobe infiltrates mimicking more common atypical community acquired pneumonia.  Occasionally the chest X-ray may appear normal, in presence of active disease and high clinical suspicion is required to order further TB investigations.^2,14^ |

**Supp Table 3** describes important clinical and radiological manifestations of tuberculosis (TB). While the working group does not currently include formal screening criteria regarding clinical or radiological evidence, some radiological findings are particularly concerning and should alert the medical director to scrutinize the potential donor very carefully.

References:

1. Davies PDO, Gordon SB, Davies G, eds. *Clinical Tuberculosis*. CRC Press; 2014. doi:10.1201/b16604

2. Heemskerk D, Caws M, Marais B, Farrar J. *Tuberculosis in Adults and Children*. http://www.springer.com/series/10138

3. Andreu J, Cáceres J, Pallisa E, Martinez-Rodriguez M. Radiological manifestations of pulmonary tuberculosis. *Eur J Radiol*. 2004;51(2):139-149. doi:10.1016/j.ejrad.2004.03.009

4. Geng E, Kreiswirth B, Burzynski J, Schluger NW. Clinical and Radiographic Correlates of Primary and Reactivation Tuberculosis. *JAMA*. 2005;293(22):2740. doi:10.1001/jama.293.22.2740

5. Kondo T, Nishiya K, Kobayashi I, et al. A case of pulmonary semi-invasive aspergillosis developing fatal acute exacerbation. *Tokai J Exp Clin Med*. 2006;31(3):91-95.

6. Ip M, Osterberg LG, Chau PY, Raffin TA. Pulmonary Melioidosis. *Chest*. 1995;108(5):1420-1424. doi:10.1378/chest.108.5.1420

7. Goodwin RA, Des Prez RM. Apical Localization of Pulmonary Tuberculosis, Chronic Pulmonary Histoplasmosis, and Progressive Massive Fibrosis of the Lung. *Chest*. 1983;83(5):801-805. doi:10.1378/chest.83.5.801

8. Bahn YS, Sun S, Heitman J, Lin X. Microbe Profile: Cryptococcus neoformans species complex. *Microbiology (N Y)*. 2020;166(9):797-799. doi:10.1099/mic.0.000973

9. Butler C, Kleinerman J. The pulmonary apical cap. *Am J Pathol*. 1970;60(2):205-216.

10. Renner RR, Pernice NJ. The apical cap. *Semin Roentgenol*. 1977;12(4):299-302. doi:10.1016/0037-198X(77)90043-8

11. Saito A, Hakamata Y, Yamada Y, et al. Pleural thickening on screening chest X-rays: a single institutional study. *Respir Res*. 2019;20(1):138. doi:10.1186/s12931-019-1116-9

12. Yousem SA. Pulmonary Apical Cap. *Am J Surg Pathol*. 2001;25(5):679-683. doi:10.1097/00000478-200105000-00018

13. McLoud T, Isler R, Novelline R, Putman C, Simeone J, Stark P. The apical cap. *American Journal of Roentgenology*. 1981;137(2):299-306. doi:10.2214/ajr.137.2.299

14. Nachiappan AC, Rahbar K, Shi X, et al. Pulmonary Tuberculosis: Role of Radiology in Diagnosis and Management. *RadioGraphics*. 2017;37(1):52-72. doi:10.1148/rg.2017160032
