# Supplementary material for: The American Association of Tissue Banks tissue donor screening for Mycobacterium tuberculosis—Recommended criteria and literature review
Source: Transpl Infect Dis. 2024 Jun 9;26(Suppl 1):e14294. doi: 10.1111/tid.14294 (PMC11578281; doi:10.1111/tid.14294)
Supplement: Supplementary file 4 — Supporting Information [file TID-26-e14294-s004.docx]

**Supp Table 4. Incidence of Tuberculosis (TB) for Foreign-born Persons Amongst Those Residing in the United States (US) for** **Various Lengths of Time**

| **Years Residing in US** | **TB Incidence** |
| --- | --- |
| 1 year or less | 121.0 |
| 1-5 years | 30.0 |
| More than 5 years | 11.9 |

**Supp Table 4** provides the incidence rate of tuberculosis (TB) which is initially present in recent immigrants to the United States (US) at various timepoints.^1^ The highest rate of TB disease development amongst immigrants is within the first several years after arrival to the US from a high-burden country, with incidence rates initially approaching the rate of their native country.^2^

References:

1. Cain KP, Haley CA, Armstrong LR, et al. Tuberculosis among Foreign-born Persons in the United States. *Am J Respir Crit Care Med*. 2007;175(1):75-79. doi:10.1164/rccm.200608-1178OC

2. McKenna MT, McCray E, Onorato I. The Epidemiology of Tuberculosis among Foreign-Born Persons in the United States, 1986 to 1993. *New England Journal of Medicine*. 1995;332(16):1071-1076. doi:10.1056/NEJM199504203321606
