# Supplementary material for: The American Association of Tissue Banks tissue donor screening for Mycobacterium tuberculosis—Recommended criteria and literature review
Source: Transpl Infect Dis. 2024 Jun 9;26(Suppl 1):e14294. doi: 10.1111/tid.14294 (PMC11578281; doi:10.1111/tid.14294)
Supplement: Supplementary file 5 — Supporting Information [file TID-26-e14294-s009.docx]

**Supp Table 5. Limitations of Using Current Year WHO-estimated Country Incidence for Donor Screening Purposes**

| **Concern** | **Specific Limitation** |
| --- | --- |
| Incidence not consistent throughout entire countries | Local demographic patterns with higher TB incidences |
|  | Isolated outbreaks of TB within otherwise low-burden countries |
| Lifetime exposure risk not reflected by current year data | Using most current year data does not reflect one’s exposure risk over a lifetime or at a past timepoint |
|  | WHO TB data are limited prior to the 1990s, and countries that now have a low TB burden (including the US) often have had a historically higher burden, reflecting modern advances in screening practices and treatment |
|  | Overall country prevalence will likely also affect lifetime exposure risk |
|  | Socioeconomic and geopolitical changes such as war, natural disasters, or pandemics may affect a region’s incidence over time |

Supp Table 5 describes the types of concerns and specific limitations acknowledged in using current year WHO-estimated country incidence for donor screening purposes.
