# Supplementary material for: The American Association of Tissue Banks tissue donor screening for Mycobacterium tuberculosis—Recommended criteria and literature review
Source: Transpl Infect Dis. 2024 Jun 9;26(Suppl 1):e14294. doi: 10.1111/tid.14294 (PMC11578281; doi:10.1111/tid.14294)
Supplement: Supplementary file 6 — Supporting Information [file TID-26-e14294-s012.docx]

**Supp Table 6. Studies Demonstrating Risk of Tuberculosis (TB) Amongst Travelers**

| **Travel Study** | **Risk and Incidence** | **Notes** |
| --- | --- | --- |
| Cobelens, 2000 ^1^ Dutch study | The risk for long-term travelers to high incidence countries overall was found to be 3.5 per 1000 person-months of travel (4200/100,000 person-years), and 2.8 per 1000 person-months of travel (3360/100,000 person-years) when only calculating for non-health-care workers. | Amounted to *annual TB incidence of 3.3%*, which was well above that of the Dutch population but similar in magnitude to that of developing countries at the time. |
| Cobelens, 2001 ^2^  Dutch study | Odds ratio for tuberculin sensitivity of 6.0 among persons who spent 3 or more months, compared to less than 3 months, in a high incidence country. |  |
| Peace Corps Volunteers Study^3^  Brown, 2016 | Peace Corps Volunteers had higher rates of active TB compared with the general US population (incidence 21.41 cases per 100 000 volunteer-years compared with 3.56 cases per 100,000 person-years in a similar age group in the US). |  |
| Dutch Study^4^  Elfrink, 2014 | TST conversion rate of 4.3 per 1000 person-months amongst travelers spending 13–52 weeks in TB-endemic countries (5112 cases per 100,000 person-years) | Pre and post travel IGRAs compared were low (0.85 per 1000 person-months), suggesting possible false positives in this study from other atypical mycobacteria, or timing differences between TST and IGRA testing, as noted by the authors. |
| New Zealand Volunteer Study^5^ Visser, 2013 | Medical records of volunteers serving abroad, and their family members, were reviewed from 1995-2011. TST conversion rate was 1.4 cases per 1000 person-months (850 cases per 100,000 person-years) |  |
| Meta-analysis ^6^  Diefenbach-Elstob, 2012 | Risk of developing LTBI varied by travel purpose and length of stay.  HCWs travelling for up to 6 months had the highest risk of developing LTBI compared to other groups travelling for up to 6 months. | Travelers observed between 1994 and 2013. Health care workers military personnel, and general travelers/volunteers |

**Supp Table 6** highlights many studies demonstrating the increased risk of TB amongst long-term travelers.

References:

1. Cobelens FG, van Deutekom H, Draayer-Jansen IW, et al. Risk of infection with Mycobacterium tuberculosis in travellers to areas of high tuberculosis endemicity. *The Lancet*. 2000;356(9228):461-465. doi:10.1016/S0140-6736(00)02554-X

2. Cobelens FGJ, van Deutekom H, Draayer‐Jansen IWE, et al. Association of Tuberculin Sensitivity in Dutch Adults with History of Travel to Areas of with a High Incidence of Tuberculosis. *Clinical Infectious Diseases*. 2001;33(3):300-304. doi:10.1086/321882

3. Brown ML, Henderson SJ, Ferguson RW, Jung P. Revisiting tuberculosis risk in Peace Corps Volunteers, 2006–13. *J Travel Med*. 2016;23(1):2-7. doi:10.1093/jtm/tav005

4. Elfrink F, van den Hoek A, Mensen ME, Sonder GJ. Screening travellers to high-endemic countries for infection with Mycobacterium tuberculosis using interferon gamma release assay; a prospective study. *BMC Infect Dis*. 2014;14(1):515. doi:10.1186/1471-2334-14-515

5. Visser JT, Edwards CA. Dengue Fever, Tuberculosis, Human Immunodeficiency Virus, and Hepatitis C Virus Conversion in a Group of Long‐Term Development Aid Workers. *J Travel Med*. 2013;20(6):361-367. doi:10.1111/jtm.12072

6. Diefenbach-Elstob TR, Alabdulkarim B, Deb-Rinker P, et al. Risk of latent and active tuberculosis infection in travellers: a systematic review and meta-analysis. *J Travel Med*. 2021;28(1). doi:10.1093/jtm/taaa214
