# Supplementary material for: The American Association of Tissue Banks tissue donor screening for Mycobacterium tuberculosis—Recommended criteria and literature review
Source: Transpl Infect Dis. 2024 Jun 9;26(Suppl 1):e14294. doi: 10.1111/tid.14294 (PMC11578281; doi:10.1111/tid.14294)
Supplement: Supplementary file 7 — Supporting Information [file TID-26-e14294-s006.docx]

**Supp Table 7. Factors that May Increase Healthcare Worker (HCW) Risk for Tuberculosis (TB) Infection**

| **Health care setting** | **Job Category involving generation of patient respiratory aerosols** | **HCW Demographics** |
| --- | --- | --- |
| Prolonged exposure to unsuspected TB cases | Bronchoscopy | Place of birth |
| Exposure in areas with poor ventilation | Endotracheal intubation and suctioning | Ethnicity |
| Patient population served (Immunocompromised/HIV, refugees, homeless) | Cough and sputum induction | Place of residence |
| Geographic location (with urban areas higher than rural and mountain areas) | Administering aerosol medications | Travel |

**Supp Table 7** highlights various healthcare workplace settings, job categories and procedures, and healthcare worker demographics that may increase the HCW’s exposure to TB infection.
