# Supplementary material for: The American Association of Tissue Banks tissue donor screening for Mycobacterium tuberculosis—Recommended criteria and literature review
Source: Transpl Infect Dis. 2024 Jun 9;26(Suppl 1):e14294. doi: 10.1111/tid.14294 (PMC11578281; doi:10.1111/tid.14294)
Supplement: Supplementary file 8 — Supporting Information [file TID-26-e14294-s008.docx]

**Supp Table 8. Immunodeficiency Factors in Chronic Kidney Disease (CKD)**

| **Cellular**  **Immunodeficiency Factors** | **Comorbid**  **Immunodeficiency Factors** |
| --- | --- |
| Chronic Underlying Inflammation^1,2^ | Comorbid immunosuppressive conditions are more likely^3–6^ |
| Hypoalbuminemia^2^ | Immunosuppressive medications in some cases (due to underlying causes or comorbid conditions) |
| Uremia^2^ | Aging^5,6^ |
| Vitamin D Deficiency^7,8^ | Malnutrition^9^ |

**Supp Table 8** provides factors leading to relative immunodeficiency in CKD.

References:

1. Kurts C, Panzer U, Anders HJ, Rees AJ. The immune system and kidney disease: basic concepts and clinical implications. *Nat Rev Immunol*. 2013;13(10):738-753. doi:10.1038/nri3523

2. Espi M, Koppe L, Fouque D, Thaunat O. Chronic Kidney Disease-Associated Immune Dysfunctions: Impact of Protein-Bound Uremic Retention Solutes on Immune Cells. *Toxins (Basel)*. 2020;12(5):300. doi:10.3390/toxins12050300

3. Koye DN, Magliano DJ, Nelson RG, Pavkov ME. The Global Epidemiology of Diabetes and Kidney Disease. *Adv Chronic Kidney Dis*. 2018;25(2):121-132. doi:10.1053/j.ackd.2017.10.011

4. Naicker S, Rahmanian S, Kopp JB. HIV and chronic kidney disease. *Clin Nephrol*. 2015;83 (2015)(S1):32-38. doi:10.5414/CNP83S032

5. Kazancioğlu R. Risk factors for chronic kidney disease: an update. *Kidney Int Suppl (2011)*. 2013;3(4):368-371. doi:10.1038/kisup.2013.79

6. Thomas MC, Cooper ME, Zimmet P. Changing epidemiology of type 2 diabetes mellitus and associated chronic kidney disease. *Nat Rev Nephrol*. 2016;12(2):73-81. doi:10.1038/nrneph.2015.173

7. Mehrotra R, Kermah DA, Salusky IB, et al. Chronic kidney disease, hypovitaminosis D, and mortality in the United States. *Kidney Int*. 2009;76(9):977-983. doi:10.1038/ki.2009.288

8. Baeke F, Gysemans C, Korf H, Mathieu C. Vitamin D insufficiency: implications for the immune system. *Pediatric Nephrology*. 2010;25(9):1597-1606. doi:10.1007/s00467-010-1452-y

9. Iorember FM. Malnutrition in Chronic Kidney Disease. *Front Pediatr*. 2018;6. doi:10.3389/fped.2018.00161
