# Supplementary material for: The American Association of Tissue Banks tissue donor screening for Mycobacterium tuberculosis—Recommended criteria and literature review
Source: Transpl Infect Dis. 2024 Jun 9;26(Suppl 1):e14294. doi: 10.1111/tid.14294 (PMC11578281; doi:10.1111/tid.14294)
Supplement: Supplementary file 9 — Supporting Information [file TID-26-e14294-s005.docx]

**Supp Table 9. Risk of Tuberculosis (TB) in Pre-dialysis Chronic Kidney Disease (CKD)**

| **Source/Study** | **Country and Dates of Data** | **Notes** | **Incidence of Tb in relation to CKD (pre-dialysis)** |
| --- | --- | --- | --- |
| Ruzangi, 2020^1^ | UK 2004-2014 | CKD stages 1 and 2 not analyzed | The incidence of TB was higher amongst patients with CKD stage 3-5 compared to those without CKD: 14.63 and 9.89 cases per 100,000 person-years.  Adjusted rate ratio was **1.42** |
| Luczynski P, 2023^2^ | Meta-analysis |  | Estimated pooled risk of TB was 57% higher in people with CKD stages 3–5 than in people without CKD  Adjusted hazard ratio: **1.57**  When stratified by CKD stage, the pooled rate of TB was highest in stages 4–5 (incidence rate ratio: **3.63**) |
| Cheng 2018^3^ | Taiwan 2000-2012 | Unable to ascertain numerical relative risk per CKD stage from published data, but can observe cumulative TB risk trend from figures | Overall incidence of pulmonary TB was 1.47-fold higher in the pre-dialysis CKD group compared to that in the non-CKD group.  Higher renal function was associated with a lower risk of TB infection and the cumulative TB risk according to CKD stage.  The risk of TB infection was highest in patients with CKD stage 5, followed by patients with CKD stage 4, stage 3, stage 2, and the risk were lowest in patients with CKD stage 1. |
| Park 2019^4^ | Korea (2012-2016) |  | Tuberculosis risk was significantly higher in the pre-dialysis CKD group.  Adjusted hazard ratio = **1.21** |
| Yan 2021^5^ | British Columbia, Canada (1996-2015) | Immigrants to Canada studied | Pre-dialysis CKD risk: **2.6**-**2.9** (univariable to multivariable analyses)   CKD on Dialysis risk: (16.2-33.2) |
| Cho 2019^6^ | Taiwan 2005-2008 | Correlation between CKD stage and TB was observed   TB risk increased by 5.1% with every 10 ml/min/1.73 m2 decrease in the estimated glomerular filtration rate | Those with Stage 1–4 CKD had a 25% increase in TB hazard than those without disease  Adjusted hazard ratio= **1.25** |

**Supp Table 9** provides an overview of literature addressing risk of TB among individuals with pre-dialysis CKD.

References:

1. Ruzangi J, Iwagami M, Smeeth L, Mangtani P, Nitsch D. The association between chronic kidney disease and tuberculosis; a comparative cohort study in England. *BMC Nephrol*. 2020;21(1):420. doi:10.1186/s12882-020-02065-4

2. Luczynski P, Holmes T, Romanowski K, et al. Risk of Tuberculosis Disease in People With Chronic Kidney Disease Without Kidney Failure: A Systematic Review and Meta-analysis. *Clinical Infectious Diseases*. 2023;77(8):1194-1200. doi:10.1093/cid/ciad364

3. Cheng KC, Liao KF, Lin CL, Liu CS, Lai SW. Chronic kidney disease correlates with increased risk of pulmonary tuberculosis before initiating renal replacement therapy. *Medicine*. 2018;97(39):e12550. doi:10.1097/MD.0000000000012550

4. Park S, Lee S, Kim Y, et al. Association of CKD with Incident Tuberculosis. *Clinical Journal of the American Society of Nephrology*. 2019;14(7):1002-1010. doi:10.2215/CJN.14471218

5. Yan M, Puyat JH, Shulha HP, Clark EG, Levin A, Johnston JC. Risk of tuberculosis associated with chronic kidney disease: a population-based analysis. *Nephrology Dialysis Transplantation*. 2021;37(1):197-198. doi:10.1093/ndt/gfab222

6. Cho PJY, Wu CY, Johnston J, Wu MY, Shu CC, Lin HH. Progression of chronic kidney disease and the risk of tuberculosis: an observational cohort study. *The International Journal of Tuberculosis and Lung Disease*. 2019;23(5):555-562. doi:10.5588/ijtld.18.0225
