# Supplementary material for: The American Association of Tissue Banks tissue donor screening for Mycobacterium tuberculosis—Recommended criteria and literature review
Source: Transpl Infect Dis. 2024 Jun 9;26(Suppl 1):e14294. doi: 10.1111/tid.14294 (PMC11578281; doi:10.1111/tid.14294)
Supplement: Supplementary file 10 — Supporting Information [file TID-26-e14294-s011.docx]

**Supp Table 10. Risk of Tuberculosis in Dialysis Patients**

| **Source** | **Date of Study Population** | **Country  of Study** | **Incidence Data** | **Highlights** |
| --- | --- | --- | --- | --- |
| Chia, 1998^1^ | 1990-1994 | British  Columbia | Age-matched relative-risk of TB 25.3 in dialysis patients.^1^ | Age-matched |
| Lundin, 1979^2^ | 1979 | US  (New York) | TB incidence in hemodialysis patients 10 times that of the general population.^2^ | Limited abstract |
| Dobler, 2011^3^ | 2001-2006 | New  Zealand | TB incidence was increased to 66.8 per 100,000 persons years after starting dialysis (incidence of culture positive TB was 50.6) vs 5.7 in general population.^3^ | Adjusted for TB incidence in country of birth, sex, age and indigenous status. Low-incidence country |
| Klote, 2006^4^ | 1995-1999 | US | Large study of dialysis population, incidence of TB was found to be 0.8% per year.^4^ | The risk was found to be constant over time |
| Ahmed, 2004^5^ | 1995-2000 | US (California) | Kaiser patients who initiated dialysis, relative risk was found to be 11.3 compared to non-dialysis patients. | Extrapulmonary disease was found in over 70% of the patients for whom the site of disease was recorded^5^ |
| Al-Efraij, 2015^6^ | Various studies from 1990-2009 | Global | Dialysis populations have a pooled unadjusted rate ratio of 7.7 (95% CI 5.9–10.0) for active TB compared with the general population. The adjusted pooled rate ratio for active TB risk decreased to 3.6 (95% CI 1.8–7.3). | Meta-analysis adjusting for age, country of origin, sex, race |

**Supp Table 10** provides an overview of studies describing relative risk, incidence, and rate ratios of active TB compared with the general population.

References:

1. Chia S, Karim M, Elwood RK, FitzGerald JM. Risk of tuberculosis in dialysis patients: a population-based study. *Int J Tuberc Lung Dis*. 1998;2(12):989-991.

2. Lundin AP, Adler AJ, Berlyne GM, Friedman EA. Tuberculosis in patients undergoing maintenance hemodialysis. *Am J Med*. 1979;67(4):597-602. doi:10.1016/0002-9343(79)90240-7

3. Dobler CC, McDonald SP, Marks GB. Risk of Tuberculosis in Dialysis Patients: A Nationwide Cohort Study. *PLoS One*. 2011;6(12):e29563. doi:10.1371/journal.pone.0029563

4. Klote MM, Agodoa LY, Abbott KC. Risk factors for Mycobacterium tuberculosis in US chronic dialysis patients. *Nephrology Dialysis Transplantation*. 2006;21(11):3287-3292. doi:10.1093/ndt/gfl488

5. Ahmed AT, Karter AJ. Tuberculosis in California dialysis patients. *Int J Tuberc Lung Dis*. 2004;8(3):341-345.

6. Al-Efraij K, Mota L, Lunny C, Schachter M, Cook V, Johnston J. Risk of active tuberculosis in chronic kidney disease: a systematic review and meta-analysis. *The International Journal of Tuberculosis and Lung Disease*. 2015;19(12):1493-1499. doi:10.5588/ijtld.15.0081
