# Supplementary material for: The American Association of Tissue Banks tissue donor screening for Mycobacterium tuberculosis—Recommended criteria and literature review
Source: Transpl Infect Dis. 2024 Jun 9;26(Suppl 1):e14294. doi: 10.1111/tid.14294 (PMC11578281; doi:10.1111/tid.14294)
Supplement: Supplementary file 11 — Supporting Information [file TID-26-e14294-s014.docx]

**Supp Table 11. Immunosuppressive Medications**

| **Corticosteroids** | **Conventional**  **DMARDs** | **Targeted synthetic DMRDs** | **Biosimilars** |
| --- | --- | --- | --- |
| Hydrocortisone  Prednisone  Prednisolone  Methylprednisolone  Dexamethasone | Methotrexate  Sulfasalazine  Hydroxychloroquine  Leflunomide  Azathioprine  Cyclosporine  Cyclophosphamide  Mycophenolate | TNF-alpha inhibitors  IL-1, IL-6, IL-17, 23 inhibitors  Anti- B-cell monoclonal antibodies | JAK inhibitors |

**Supp Table 11** provides categories of immunosuppressive medications and examples of medications within each category.
