# Supplementary material for: The American Association of Tissue Banks tissue donor screening for Mycobacterium tuberculosis—Recommended criteria and literature review
Source: Transpl Infect Dis. 2024 Jun 9;26(Suppl 1):e14294. doi: 10.1111/tid.14294 (PMC11578281; doi:10.1111/tid.14294)
Supplement: Supplementary file 12 — Supporting Information [file TID-26-e14294-s001.docx]

**Supp Table 12. Relationship Between Immunosuppressive Medications and Tuberculosis (TB)**

| **Immunomodulatory Agent** | **Review** |
| --- | --- |
| Corticosteroids | For patients who are treated with corticosteroids, the risk of TB reactivation increases 2.8 -7.7-fold.^1^ Among a cohort of 24,282 patients with RA in Quebec, 18% of those with tuberculosis were current glucocorticoid users compared to 8% for controls (p=0.03). The RR of TB was 2.4 (95% CI 1.1, 5.4) with use of corticosteroids.^2^ European Crohn’s and Colitis Organization (ECCO), and American Thoracic Society (ATS), and the U.S. Centers for Disease Control and Prevention (CDC) guidelines recommend the treatment of latent TB infection (LTBI) in patients receiving corticosteroids at a dose equivalent to prednisolone 15 mg/day for at least 1 month.^3,4^ |
| Methotrexate | The added risk of TB reactivation is low with methotrexate therapy. An increased risk of TB reactivation has been reported only when methotrexate was used in combination with anti-TNF agents^5^ or glucocorticoids.^6^ |
| 5-aminosalicylates and their pro-drug, sulfasalazine | The safety profile of 5-ASAs was evaluated in large scale cohort studies; no increased risk of major or opportunistic infections was identified.^7^ No association between 5-ASA use and TB reactivation has been reported. |
| Thiopurines (cytotoxic purine analogues including mercaptopurine and azathioprine) | Thiopurines impair adaptive immune responses^8^. A recent systematic review of 40 randomized trials found the highest risk of TB reactivation with combination therapy compared to controls (OR 54; 95% CI 5.3–88)^9^. |
| Calcineurin inhibitors  Cyclosporine  Tacrolimus | The calcineurin inhibitors (CNI) cyclosporine and tacrolimus are commonly used as a component of immunosuppression after organ or stem cell transplantation with wide variation in doses and combinations.^10^ CNI increases the risk of active TB in post-transplant patients.^11^ A retrospective study reported 2.5 times greater (p = 0.0311) risk of TB reactivation early post-transplant with cyclosporine compared to a combination of corticosteroids and azathioprine (which is uncommonly used at present).^12^ The risk of CNI-related serious and opportunistic infections in non-transplant settings is lower,^13^ depending on dosing and concomitant immunosuppressive agents. For most rheumatological and dermatological conditions is CNI doses are generally much lower (2.5–5 mg/ kg) the doses used in transplantation. |
| Tumor Necrosis Factor alpha inhibitors  Adalimumab  Certolizumab  Golimumab  Infliximab  Etanercept | Use of TNF alpha inhibitors is associated with increased risk of TB reactivation, reinfection, and dissemination to extra-pulmonary sites. ^9^*^,^*^14^ TNF inhibitors also carry the risk of seroconversion during the treatment. In patients with RA treated with TNF inhibitors there is a 4 - 8-fold increased risk of active TB.^15^ Risk is greatest in countries with high endemnicity.^16^ The soluble receptor of TNF fusion protein (etanercept) has a lower risk than anti -TNF monoclonal antibodies.^17,18^ TB screening (TST or IGRA) and therapy of latent TB are recommended prior to use of TNF inhibitors and other biologic agents.^19^ |
| Anti-Interleukin (IL) 6 receptor antibody  Tocilizumab | 8 active TB cases were reported in 21 trials of patients with RA treated with anti-IL-6 Tocilizumab, largely in countries with high TB risk.^20^ The risk for TB activation with more prolonged IL-6 inhibitor or IL-6 receptor antagonist therapies seem modest but require further study.^21–24^ |
| Anti-Interleukin (IL)-12/23 antibody  Ustekinumab | The Psoriasis Longitudinal Assessment and Registry (PSOLAR) study of 3474 patients with psoriasis and Psoriatic arthritis treated with Ustekinumab revealed no TB cases with a median follow-up of 1.60 years.^25^ Ustekinumab has limited risk for TB reactivation in the short term.^21,22,24^ |
| Anti-Interleukin (IL)-17, Secukinumab  Ixekizumab  Brodalumab | To date, there is no evidence that mycobacterial immune response is negatively affected by monoclonal antibodies targeting IL-17A.^22,26^ Limited clinicals trials and observational studies found no increased risk of tuberculosis infection.^21,24,27,28^ |
| Anti-Interleukin (IL) 23, Guselkumab  Risankizumab  Tildrakizumab | In clinical trials with use of anti-IL 23 therapies the participants were screened for LTBI and received prophylactic anti-TB treatment in most cases; possibly as a result, no cases of TB were reported.^29^ |
| Anti-CD20-antibody  Rituximab | CD-20 is found predominantly on the surface of mature B-cells and so anti-CD20-antibody therapy does not affect T- cell mediated immune response to TB. There is no evidence of an increased incidence of tuberculosis with rituximab therapy: only two cases of active TB have been reported in RA patients during a follow-up time of 9.5 years.^30^ No cases of active TB were reported with anti-CD20-antibody therapy in countries with a high incidence of tuberculosis.^21,31^ |
| Janus kinase (JAK) inhibitor  Tofacitinib,  Baricitinib,  Upadacitinib,  Filgotinib | JAK kinase inhibitors (Jakinibs) target four members – JAK1, JAK2, JAK3 and TYK2. The immunomodulating effect on the immune response against mycobacteria is comparable to that of TNF blockers. ^21,22,32,33^. Opportunistic infections are common in recipients of these agents after organ transplantation and TB screening is required before use. TB was most frequent in countries with a high TB incidence.^16^ Many TB cases after JAK inhibitor therapy are extrapulmonary without classical symptoms of fever, cough, and weight loss. In the extended observation period of the phase II and III trials of tofacitinib, risk increase was modest with 26 cases in 5671 patients, primarily in high-risk environments.^16^ |

**Supp Table 12** provides an overview of immunosuppressive medications’ known impact on MTB. Residence in regions endemic for tuberculosis or travel to such areas will increase the risk of latent or active TB in all individuals regardless of immune status.

References:

1. Jick SS, Lieberman ES, Rahman MU, Choi HK. Glucocorticoid use, other associated factors, and the risk of tuberculosis. *Arthritis Rheum*. 2006;55(1):19-26. doi:10.1002/art.21705

2. Brassard P, Lowe A, Bernatsky S, Kezouh A, Suissa S. Rheumatoid arthritis, its treatments, and the risk of tuberculosis in Quebec, Canada. *Arthritis Care Res (Hoboken)*. 2009;61(3):300-304. doi:10.1002/art.24476

3. ATS/CDC Statement Committee on Latent Tuberculosis Infection. Targeted Tuberculin Testing and Treatment of Latent Tuberculosis Infection. *MMWR*. 2000;49(RR-6):1-51.

4. Kucharzik T, Ellul P, Greuter T, et al. ECCO Guidelines on the Prevention, Diagnosis, and Management of Infections in Inflammatory Bowel Disease. *J Crohns Colitis*. 2021;15(6):879-913. doi:10.1093/ecco-jcc/jjab052

5. Salliot C, van der Heijde D. Long-term safety of methotrexate monotherapy in patients with rheumatoid arthritis: a systematic literature research. *Ann Rheum Dis*. 2009;68(7):1100-1104. doi:10.1136/ard.2008.093690

6. Fragoulis GE, Constantinou CA, Sipsas N V, Hyrich KL, Nikiphorou E. Tuberculosis in inflammatory arthritis: are biological therapies the only culprits? *Lancet Rheumatol*. 2019;1(3):e138-e140. doi:10.1016/S2665-9913(19)30012-8

7. Ransford RAJ. Sulphasalazine and mesalazine: serious adverse reactions re-evaluated on the basis of suspected adverse reaction reports to the Committee on Safety of Medicines. *Gut*. 2002;51(4):536-539. doi:10.1136/gut.51.4.536

8. Pettit AR. Mechanism of action of purine analogues in chronic lymphocytic leukemia. *British Journal Haematology*. 2003;121:692-702.

9. Lorenzetti R, Zullo A, Ridola L, et al. Higher risk of tuberculosis reactivation when anti-TNF is combined with immunosuppressive agents: A systematic review of randomized controlled trials. *Ann Med*. 2014;46(7):547-554. doi:10.3109/07853890.2014.941919

10. Lichtiger S, Present DH, Kornbluth A, et al. Cyclosporine in Severe Ulcerative Colitis Refractory to Steroid Therapy. *New England Journal of Medicine*. 1994;330(26):1841-1845. doi:10.1056/NEJM199406303302601

11. Agarwal SK, Bhowmik D, Mahajan S, Bagchi S. Impact of type of calcineurin inhibitor on post‐transplant tuberculosis: Single‐center study from India. *Transplant Infectious Disease*. 2017;19(1). doi:10.1111/tid.12626

12. John GT, Shankar V, Abraham AM, Mukundan U, Thomas PP, Jacob CK. Risk factors for post-transplant tuberculosis. *Kidney Int*. 2001;60(3):1148-1153. doi:10.1046/j.1523-1755.2001.0600031148.x

13. Schneeweiss MC, Perez-Chada L, Merola JF. Comparative safety of systemic immunomodulatory medications in adults with atopic dermatitis. *J Am Acad Dermatol*. 2021;85(2):321-329. doi:10.1016/j.jaad.2019.05.073

14. Dixon WG, Watson K, Lunt M, Hyrich KL, Silman AJ, Symmons DPM. Rates of serious infection, including site‐specific and bacterial intracellular infection, in rheumatoid arthritis patients receiving anti–tumor necrosis factor therapy: Results from the British Society for Rheumatology Biologics Register. *Arthritis Rheum*. 2006;54(8):2368-2376. doi:10.1002/art.21978

15. Winthrop K, Baxter R, Liu L, et al. Mycobacterial diseases and antitumour necrosis factor therapy in USA. *Ann Rheum Dis*. 2013;72(1):37-42. doi:10.1136/annrheumdis-2011-200690

16. Winthrop KL, Park SH, Gul A, et al. Tuberculosis and other opportunistic infections in tofacitinib-treated patients with rheumatoid arthritis. *Ann Rheum Dis*. 2016;75(6):1133-1138. doi:10.1136/annrheumdis-2015-207319

17. Minozzi S, Bonovas S, Lytras T, et al. Risk of infections using anti-TNF agents in rheumatoid arthritis, psoriatic arthritis, and ankylosing spondylitis: a systematic review and meta-analysis. *Expert Opin Drug Saf*. 2016;15(sup1):11-34. doi:10.1080/14740338.2016.1240783

18. Dixon WG, Hyrich KL, Watson KD, et al. Drug-specific risk of tuberculosis in patients with rheumatoid arthritis treated with anti-TNF therapy: Results from the British Society for Rheumatology Biologics Register (BSRBR). *Ann Rheum Dis*. 2010;69(3):522-528. doi:10.1136/ard.2009.118935

19. Arkema E V, Jonsson J, Baecklund E, Bruchfeld J, Feltelius N, Askling J. Are patients with rheumatoid arthritis still at an increased risk of tuberculosis and what is the role of biological treatments? *Ann Rheum Dis*. 2015;74(6):1212-1217. doi:10.1136/annrheumdis-2013-204960

20. Schiff MH, Kremer JM, Jahreis A, Vernon E, Isaacs JD, van Vollenhoven RF. Integrated safety in tocilizumab clinical trials. *Arthritis Res Ther*. 2011;13(5):R141. doi:10.1186/ar3455

21. Evangelatos G, Koulouri V, Iliopoulos A, Fragoulis GE. Tuberculosis and targeted synthetic or biologic DMARDs, beyond tumor necrosis factor inhibitors. *Ther Adv Musculoskelet Dis*. 2020;12. doi:10.1177/1759720X20930116

22. Winthrop KL, Mariette X, Silva JT, et al. ESCMID Study Group for Infections in Compromised Hosts (ESGICH) Consensus Document on the safety of targeted and biological therapies: an infectious diseases perspective (Soluble immune effector molecules [II]: agents targeting interleukins, immunoglobulins and complement factors). *Clinical Microbiology and Infection*. 2018;24:S21-S40. doi:10.1016/j.cmi.2018.02.002

23. Cantini F, Nannini C, Niccoli L, et al. Guidance for the management of patients with latent tuberculosis infection requiring biologic therapy in rheumatology and dermatology clinical practice. *Autoimmun Rev*. 2015;14(6):503-509. doi:10.1016/j.autrev.2015.01.011

24. Cantini F, Niccoli L, Capone A, Petrone L, Goletti D. Risk of tuberculosis reactivation associated with traditional disease modifying anti-rheumatic drugs and non-anti-tumor necrosis factor biologics in patients with rheumatic disorders and suggestion for clinical practice. *Expert Opin Drug Saf*. 2019;18(5):415-425. doi:10.1080/14740338.2019.1612872

25. Kalb RE, Fiorentino DF, Lebwohl MG, et al. Risk of Serious Infection With Biologic and Systemic Treatment of Psoriasis. *JAMA Dermatol*. 2015;151(9):961. doi:10.1001/jamadermatol.2015.0718

26. Elewski BE, Baddley JW, Deodhar AA, et al. Association of Secukinumab Treatment With Tuberculosis Reactivation in Patients With Psoriasis, Psoriatic Arthritis, or Ankylosing Spondylitis. *JAMA Dermatol*. 2021;157(1):43. doi:10.1001/jamadermatol.2020.3257

27. Reich K, Gooderham M, Green L, et al. The efficacy and safety of apremilast, etanercept and placebo in patients with moderate‐to‐severe plaque psoriasis: 52‐week results from a phase IIIb, randomized, placebo‐controlled trial (LIBERATE). *Journal of the European Academy of Dermatology and Venereology*. 2017;31(3):507-517. doi:10.1111/jdv.14015

28. Ribero S, Licciardello M, Quaglino P, Dapavo P. Efficacy and Safety of Secukinumab in Patients with Plaque Psoriasis and Latent Tuberculosis. *Case Rep Dermatol*. 2019;11(Suppl. 1):23-28. doi:10.1159/000501989

29. Nogueira M, Warren RB, Torres T. Risk of tuberculosis reactivation with interleukin (IL)‐17 and IL‐23 inhibitors in psoriasis – time for a paradigm change. *Journal of the European Academy of Dermatology and Venereology*. 2021;35(4):824-834. doi:10.1111/jdv.16866

30. Van Vollenhoven RF, Fleischmann RM, Furst DE, Lacey S, Lehane PB. Longterm safety of rituximab: Final report of the rheumatoid arthritis global clinical trial program over 11 years. *Journal of Rheumatology*. 2015;42(10):1761-1766. doi:10.3899/jrheum.150051

31. Mikulska M, Lanini S, Gudiol C, et al. ESCMID Study Group for Infections in Compromised Hosts (ESGICH) Consensus Document on the safety of targeted and biological therapies: an infectious diseases perspective (Agents targeting lymphoid cells surface antigens [I]: CD19, CD20 and CD52). *Clinical Microbiology and Infection*. 2018;24:S71-S82. doi:10.1016/j.cmi.2018.02.003

32. Cantini F, Blandizzi C, Niccoli L, Petrone L, Goletti D. Systematic review on tuberculosis risk in patients with rheumatoid arthritis receiving inhibitors of Janus Kinases. *Expert Opin Drug Saf*. 2020;19(7):861-872. doi:10.1080/14740338.2020.1774550

33. Wollenhaupt J, Lee EB, Curtis JR, et al. Safety and efficacy of tofacitinib for up to 9.5 years in the treatment of rheumatoid arthritis: final results of a global, open-label, long-term extension study. *Arthritis Res Ther*. 2019;21(1):89. doi:10.1186/s13075-019-1866-2
