# Supplementary material for: The American Association of Tissue Banks tissue donor screening for Mycobacterium tuberculosis—Recommended criteria and literature review
Source: Transpl Infect Dis. 2024 Jun 9;26(Suppl 1):e14294. doi: 10.1111/tid.14294 (PMC11578281; doi:10.1111/tid.14294)
Supplement: Supplementary file 13 — Supporting Information [file TID-26-e14294-s002.docx]

**Supp Table 13. Factors Complicating the Ability to Detect or Diagnose Tuberculosis (TB) in Individuals > 65 years old**

| **Factor** | **Resulting Concern for Detection/Diagnosis** |
| --- | --- |
| Immune senescence | Declining immune function affecting both adaptive and innate immunity   - Decreases ability to activate immunity to past exposures^2,3^ - In Testing (by tuberculin skin test (TST) or interferon-gamma release assay (IGRA)), older age increases the odds of false-negative results (anergy, immune suppression)^1,2^ |
| Increased risk of extra-pulmonary disease and atypical presentation | TB is often overlooked on the differential diagnosis when there is no obvious pulmonary involvement   - only 30%-65% of cases of miliary TB are diagnosed based on sputum culture ^2,3^ |
| Increased risk of atypical or nonspecific symptom presentation (e.g., fewer presenting with cough, fever, or hemoptysis)   - more frequent presentation with dyspnea, weakness, weight loss, or mental status changes; radiologic findings of upper lobe or cavitary involvement is less frequent - pulmonary involvement with more frequent negative sputum examination - more frequent extrapulmonary disease | Non-specific symptoms or symptoms presenting differently than expected can delay or stymie diagnosis.^1–4^  Misattribution of symptoms to more common causes may occur during hospitalization prior to death, and be carried forward for post-mortem review of donor eligibility.   - For example, infiltrates attributed to community-acquired pneumonia or heart failure, nodules attributed to cancer. |
| Increased frequency and risk for co-morbid conditions:   - frailty - nutritional deficiencies - malignancy - inflammatory diseases - autoimmune diseases - diabetes - chronic kidney disease | Co-morbidities increase likelihood of TB disease by impacting the immune system, may include use of immune modulating drugs for treatment, and may mask or delay clinical suspicion of the presence of TB^1,2,4^ |

**Supp Table 13** provides a list of multiple factors that occur in individuals 65 and older that increases their risk for TB infection (TBI) and TB disease (TB), and decreases the ability to recognize the presence of TBI or TB.

References:

1. Wu IL, Chitnis AS, Jaganath D. A narrative review of tuberculosis in the United States among persons aged 65 years and older. *J Clin Tuberc Other Mycobact Dis*. 2022;28:100321. doi:10.1016/j.jctube.2022.100321

2. Khan A, Rebhan A, Seminara D, Szerszen A. Enduring Challenge of Latent Tuberculosis in Older Nursing Home Residents: A Brief Review. *J Clin Med Res*. 2019;11(6):385-390. doi:10.14740/jocmr3763

3. Caraux-Paz P, Diamantis S, de Wazières B, Gallien S. Tuberculosis in the Elderly. *J Clin Med*. 2021;10(24):5888. doi:10.3390/jcm10245888

4. Agarwal K. Failure to thrive in older adults: Evaluation. *UpToDate*. Published online June 29, 2021. Accessed January 22, 2024. https://www.uptodate.com/contents/failure-to-thrive-in-older-adults-evaluation?search=Failure%20to%20thrive%20in%20older%20adults:%20Evaluation&source=search_result&selectedTitle=1~150&usage_type=default&display_rank=1
