# Supplementary material for: The American Association of Tissue Banks tissue donor screening for Mycobacterium tuberculosis—Recommended criteria and literature review
Source: Transpl Infect Dis. 2024 Jun 9;26(Suppl 1):e14294. doi: 10.1111/tid.14294 (PMC11578281; doi:10.1111/tid.14294)
Supplement: Supplementary file 14 — Supporting Information [file TID-26-e14294-s013.docx]

**Supp Table 14. IGRA Specimen Handling and Testing: Comparison of Key Factors Impacting Utility in Tissue Donors**

|  | **QuantiFERON-TB Gold Plus^1,2^ (QF)** | **TSpot.TB^3^ (TS)** | **Comments** |
| --- | --- | --- | --- |
| **Workflow** | 1. Sample Collection 2. Incubation 3. Storage (optional) 4. Measure IFN-g concentration   Possible results: Positive, negative, indeterminate | 1. Sample Collection 2. Processing (centrifugation + cell separation) 3. Storage (optional) 4. Incubation 5. Measure number of IFN-g producing cells (spots)   Possible results: Positive, negative, borderline, invalid | Indeterminate results are common and cannot be interpreted to adjudicate donor disposition. |
| **Intended Use** | The LIAISON® QuantiFERON®-TB Gold Plus assay is an *in vitro* diagnostic test for the detection of interferon-γ (IFN-γ) in human lithium heparin plasma by chemiluminescence immunoassay (CLIA) using the LIAISON® XL Analyzer. QIAGEN QuantiFERON®-TB Gold Plus Blood Collection Tubes, containing a peptide cocktail simulating ESAT-6 and CFP-10 proteins, are used in conjunction with the LIAISON® QuantiFERON®-TB Gold Plus assay to stimulate cells in heparinized whole blood. Detection of IFN-γ _is used to identify *in vitro* responses to these peptide antigens that are associated with *Mycobacterium tuberculosis* infection.  The assay is a qualitative indirect test for *M. tuberculosis* infection (including disease) and **is intended for use in conjunction with risk assessment, radiography, and other medical and diagnostic evaluations to assist the clinician in making individual patient management decisions.** The LIAISON® QuantiFERON®-TB Gold Plus assay must be performed using the LIAISON® XL Analyzer. | The T-SPOT®TB test is an in vitro diagnostic test for the detection of effector T cells that respond to stimulation by Mycobacterium tuberculosis antigens ESAT-6 and CFP 10 by capturing interferon gamma (IFN-γ) in the vicinity of T cells in human whole blood collected in sodium citrate or sodium or lithium heparin. It is intended for use as an aid in the diagnosis of M. tuberculosis infection.  The T-SPOT.TB test is an indirect test for M. tuberculosis infection (including disease) and is **intended for use in conjunction with risk assessment, radiography, and other medical and diagnostic evaluations.** | **Specimens from deceased donors (“cadaveric” specimens) are not validated for these assays.** These assays are intended for use in conjunction with medical evaluation which cannot be performed in deceased individuals. |
| **Sample Type** | Whole blood collected in anticoagulant tubes | Whole blood collected in anticoagulant tubes | Whole blood is collected for each assay; peripheral blood mononuclear cells are separated for processing (TS) |
| **Collection tube** | 1) LIAISON® QuantiFERON®-TB Gold Plus assay must be performed using lithium heparin plasma from whole blood samples collected, handled and processed with QIAGEN QuantiFERON®-TB Gold Plus (QFT-Plus) Blood Collection Tubes (BCTs)  2) into lithium- or sodium-heparin tube for up to 16 hours storage prior to transfer to QFT-Plus BCT and incubation^†^ | 1) Without T-Cell Xtend reagent: sodium citrate or sodium heparin CPT, with PBMCs separated in the tube  2) With T-cell Xtend reagent: lithium heparin tubes only (advises CPT tubes and EDTA tubes should NOT be used) | Requires use of proprietary collection tubes (QF) |
| **Sample Volume** | For QFT-Plus, 1 mL (range 0.8-1.2 mL)  For a lithium- or sodium-heparin BCT, minimum volume is 5 mL | Adults and children 10 years old and over^‡^: one 8mL or two 4mL CPT tubes or one lithium-heparin 6mL tube.  A patient’s cells can be pooled^§^, if necessary to obtain sufficient cells from multiple tubes of blood which were collected and processed concurrently. | Sample availability (unclotted blood and adequate cell numbers) may be limiting for deceased donors |
| **Storage conditions prior to processing (TS) or incubation (QF)** | QFT-Plus BCT: hold at 17-25°C, total time from blood draw to 37°C incubation must not exceed 16 hours | Do not refrigerate or freeze. Storage temps prior to processing (incubation) 18-25 °C. | Total elapsed time until sample processing (TS) or incubation (QF) step (i.e., *steps that require the sample to be in a laboratory*) ranges from 8-16 hours if samples are stored at 17-25°C (QF) or 18-25°C, and up to 53 hours if sample is stored at 2-8°C (QF).  For specimens collected after death, times should be calculated from the TIME OF DEATH rather than from time of specimen collection. These assays measure T-cell activity and require viable cells. |
|  | Lithium- or sodium-heparin tube: Total time from blood draw to 37°C incubation must not exceed 16 hours, or 2) hold at 2-8°C total time from blood draw to 37°C incubation must not exceed 53 hours. | Without use of T-cell Xtend reagent: blood samples should be processed (centrifuged and cells separated) within 8 hours of collection, with testing is performed subsequently (without delay).  With use of T-cell Xtend reagent: addition of T-cell Xtend reagent must occur just prior to processing (centrifugation and cell separation), to allow sample storage between processing and testing to extend to up to 32 hours—but processing must occur as soon as feasible but not longer than 8 hours after collection. |  |
| **Necessary sample handling steps to complete prior to testing** | For testing performed directly from centrifuged QIAGEN QFT-Plus BCT, plasma samples can be stored in the incubated & centrifuged blood collection tubes for up to 28 days at 2°-8°C prior to testing. Plasma samples transferred from QFT-Plus Blood Collection Tubes to secondary storage tubes can be stored at 2°-8°C for up to 28 days prior to testing, or stored frozen at –20°C for up to six months prior to testing. Frozen plasma samples, once thawed, should be mixed well before testing Frozen plasma samples remain stable for up to 4 freeze/thaw cycles. | The extension of time afforded by use of T-cell Xtend reagent only extends the time between centrifugation and cell separation and the incubation step. Once the incubation step is performed, cells must be counted to ensure sufficient PBMCs and then placed on the instrument for 16–20-hour incubation; ~3 hours required to perform development steps and read the plates. |  |

^†^ Whole blood samples must be collected and processed in accordance with the QIAGEN QuantiFERON®-TB Gold Plus Blood Collection Tubes instructions for use^2^.

^‡^ Instructions are also provided for children between the ages of 2-10, and for children aged less than 2

^§^ Sample volume guidelines provided are for immunocompetent patients, and IFU notes that additional sample volume may be required if sufficient cells are not obtained, and stipulates that a patient’s cells can be pooled, if necessary to obtain sufficient cells from multiple tubes of blood

**Supp Table 14** provides key Interferon-gamma release assay (IGRA) specimen and testing requirements impacting ability to perform in tissue donors are provided for the two assays approved in the United States, and commentary regarding challenges faced in the deceased donor setting are provided.

References:

1. DiaSorin Inc. LIAISON QuantiFERON-TB Gold Plus ([REF] 311020). *Instructions for Use*. Published online 2019. Accessed February 5, 2024. https://www.accessdata.fda.gov/cdrh_docs/pdf18/P180047D.pdf

2. Qiagen. QuantiFERON® -TB Gold Plus Blood Collection Tubes. *Instructions for Use (Version 1)*. Published online September 2023. Accessed February 5, 2024. https://www.qiagen.com/us/resources/download.aspx?id=22bdba7c-4b2b-44cc-9a1c-715d264f87a0&lang=en

3. Oxford Immunotec. T-SPOT.TB. *Instructions for Use [PI-TB-US-0001 V8]*. Accessed February 5, 2024. https://www.tspot.com/wp-content/uploads/2020/09/TB-PI-US-0001-V8.pdf
