# Supplementary material for: The American Association of Tissue Banks tissue donor screening for Mycobacterium tuberculosis—Recommended criteria and literature review
Source: Transpl Infect Dis. 2024 Jun 9;26(Suppl 1):e14294. doi: 10.1111/tid.14294 (PMC11578281; doi:10.1111/tid.14294)
Supplement: Supplementary file 15 — Supporting Information [file TID-26-e14294-s015.docx]

**Supp Table 15. Tuberculosis (TB) Risk Factor Criteria and Working Group (WG) Commentary**

| **Criterion** | **Expert Commentary** |
| --- | --- |
| Individuals with a history (ever) of tuberculosis disease (sometimes referred to as “active” or “clinically active” tuberculosis) | Individuals known to be infected with TB and develop TB disease should not donate human tissues for transplantation because TB disease requires prolonged therapy (months); documentation of adequate compliance is difficult and unlikely to be reliably available/verifiable at the time of donor screening. If treatment is inadequate, there is a possibility of relapse of TB disease. |
| Individuals with a history of (latent) tuberculosis infection initially diagnosed within the past two (2) years (i.e., the individual has had a positive test for tuberculosis)^†^  or greater than two (2) years prior to donation | The highest risk of reactivation is within the first two years after infection/positive test and therefore donors with this history should be ineligible to donate tissues.  Although the risk for MTB transmission by a latent infection is certainly less than that from active disease, tissues processed in a manner to retain viable cells must be screened with a higher level of caution than those that are more highly processed. Therefore, those with known remote latent infection should be excluded from donating tissues containing viable cells. |
| Age > 65 | Data show that disease reactivation is more likely to occur in persons older than 65, and that TB is more difficult to diagnose in that age group due to immune senescence and accumulated comorbidities. |
| Individuals who, within the past 2 years, traveled for >3 months or immigrated from a country with most current available tuberculosis incidence of > 20 (rate per 100,000 population) available on WHO TB country profile website: <https://worldhealthorg.shinyapps.io/tb_profiles/> | The WG determined tissue donor suitability screening should consider any cumulative travel to or residence in a high TB burden country > 3 months, in addition to a donor’s country of birth, as increased TB exposure risks.  The selected threshold for TB incidence rate of 20 per 100,000 population aligns with the CDC recommendations and is useful as a screening threshold for high burden country of birth and travel. In addition, the threshold may be considered in stratifying additional risk factors for TB exposure, although available data regarding other exposures were not as clear or consistently available as TB incidence by country.  Recent (within the past 2 years) immigration from, or prolonged travel to, countries with high TB incidence countries carries a higher chance of disease progression^1^ and donors with this risk factor should be excluded from donating viable cell tissues. |
| Exposure to an individual with tuberculosis disease within the past 2 years | TB disease occurs most frequently within 2 years post-infection. There is no known safe time of exposure to someone with active TB. |
| Persons experiencing homelessness housed in shelters or other congregate setting, <2 years ago | It is best to request detailed history regarding the timeline of homelessness, the duration of homelessness, residing homeless locations, and any known exposure to active TB.  Perhaps, PEH who reside in isolated areas, cars, or with rotating members of extended family will not have such a significant exposure risk but circumstances can be highly variable to draw a certain conclusion.  WG determined that homelessness that includes being housed in shelters or other congregate settings, for any period, is a significant exposure risk factor. Those housed in congregate settings in the last 2 years are at highest risk for TB disease, and should not donate viable cell tissues. |
| Incarceration <2 years ago | WG concludes that incarceration constitutes a considerable exposure risk and should be carefully evaluated during tissue donor screening, especially for tissues containing viable cells. |
| ESRD (CKD 5) with or without dialysis | The WG determined that immune suppression and subsequent reactivation risk in this population is well-described in the literature and is likely the main TB risk factor in ESRD and dialysis patients. Persons with chronic renal failure, ESRD, or CKD Stage 5 with or without initiation of dialysis should be excluded from donating tissues containing viable cells. |
| Solid organ transplant (SOT) recipients | Given the increased incidence, disease severity and probability of dissemination, organ transplant recipients represent a high-risk category for TB in tissue donation and the WG recommends excluding them from viable cell donation. |
| Exposure risks occurring >2 years ago including persons who:  were born in or traveled for >3 months or immigrated from a country with most current available tuberculosis incidence of > 20 (rate per 100,000 population)  were exposed to an individual with tuberculosis disease  ever experienced homelessness housed in shelters or other congregate setting  were ever incarcerated | WG concludes these exposures constitute a considerable risk and should be carefully evaluated during tissue donor screening, especially for tissues containing viable cells. Risks factors for exposure which occurred more than 2 years ago have less of a chance of progressing to tuberculosis disease.    If a remote exposure risk factor is present along with any risk factor for reactivation, donors should be excluded from donating tissues containing viable cells. |
| Advanced kidney disease, pre-dialysis—otherwise known as CKD Stage 4, GFR < 30 | Though the risk is still somewhat low independently, when combined with various TB exposure factors, it is reasonable to assume the total TB reactivation risk will be higher due to immune suppression. WG recommends that advanced kidney disease, pre-dialysis (CKD Stage 4, GFR < 30) should be accounted for as a reactivation risk in those who have exposure risk factors to TB. |
| Diabetes mellitus (DM) | Data are clear that DM is a reactivation risk factor in individuals previously exposed to TB, and the WG included a *medical diagnosis* of DM as part of the recommended criteria. |
| Cirrhosis or alcoholic liver disease | Given the increased incidence of TB compared to general population and frequent EPTB, WG consider liver cirrhosis as an independent risk factor for TB reactivation in individuals previously exposed to TB, and included as part of the recommendation criteria when screening donors of tissues containing viable cells. |
| Alcohol use disorder (AUD) / excessive or heavy alcohol use | WG concluded that AUD is an independent risk factor for TB reactivation—previously referred to with terms like alcohol abuse, excessive alcohol use, heavy alcohol use. The application of these data in the donor setting is challenging given that quantification of alcohol intake is often not available for deceased donors and AUD may not be formally diagnosed in available medical records. For the purposes of donor screening criteria, a medical diagnosis of AUD, the presence of alcohol related cirrhosis or records indicating heavy alcohol use documented by health providers or family members, may be used as a surrogate marker for clinically significant AUD to inform donor selection. |
| Use of immunosuppressive drugs | Immune system function is necessary to contain MTB. Many immunosuppressive drugs have a direct association with TB risk, some black box warnings. Newer immunosuppressive medications have not been in use for a sufficient time to draw conclusions on association with TB risk. |
| Items reviewed but not included in exclusion criteria – but are useful to consider on a case-by-case basis | Our review did not support considering prison workers as being at a similarly high risk of exposure as incarcerated persons (IPs).  Available data indicate TB risk in HCWs correlates with the community in which they reside rather than with their job. The WG does not recommend HCW exclusion from donation due to TB risk if no other risk factors are present.  Although historical TB incidence data in long term care (LTC) residents is concerning, no evidence exists indicating current significant nursing home TB outbreaks. WG already recommends age-based exclusion for donation of tissues containing viable cells, and determined there is inadequate evidence to exclude younger LTC residents from donation.  HSC is associated with an increased risk of TB compared to the general population, although occurring less frequently than in SOT recipients. Primary hematological disorders are also associated with a higher incidence of MTB infections, but are not anticipated to be accepted as tissue donors.  HSC transplant recipients represent a risk for TB reactivation, particularly while on immunosuppressive medications (criteria already exists) or before their underlying malignancy is cured.  Primary hematological disorders themselves are also associated with a higher incidence of MTB infections, but are not anticipated to be accepted as tissue donors while undergoing treatment. Data do not exist to adequately inform a length of time after HSC when that individual is cured enough to not have immune system impact, or more importantly, when the risk for TB reactivation wanes. |
| Additional items to consider that are not included in the criteria | Silicosis: Historically, silicosis has been associated with increased risk of active TB. Through recent workplace safety and TB screening initiatives,^2^ and in combination with the current low baseline TB rate in the US, silicosis-tuberculosis is now much rarer.^3,4^  Persons with silicosis should be evaluated for exposure risk factors to TB, especially if the occupational exposure for silicosis occurred in a country with a high TB burden or there are other exposure risk factors for TB. Caution should be used when evaluating persons with silicosis for viable cell tissues.  Malnutrition: Malnutrition should be considered on a case-by-case basis, as nutritional deficiencies can have a profound effect on the immune system and the ability to prevent reactivation of latent TB.   Other acute or chronic immunosuppression: Any condition not listed above that is found to have a deleterious effect on a person’s immune status should be taken into consideration when combined with TB exposure risk factors during evaluation for donation of viable cell tissues.   There are likely to be occupational or social-demographic risk factors that are not captured in generalized reported data, including but not limited to local outbreaks or clusters of TB, random exposure encounters in crowded areas, family or friends visiting from high burden regions, occupational exposures (which were of higher risk in the distant past), or mycobacteria laboratory work. Because the risk of exposure to TB in any setting is not zero, medical directors should err on the side of caution when evaluating suitability of viable cell tissue donation when there is suspicion for TB disease based on clinical and/or epidemiological data available for the donor. |

^†^ Tests for tuberculosis include TB skin test (other names used interchangeably—TB Skin Test (TST), Purified Protein Derivative (PPD) or Mantoux) and Interferon Gamma Release Assay (IGRA) blood tests (e.g., QuantiFERON-TB Gold, T-SPOT).

**Supp Table 15** provides expert commentary regarding the reasons for selection or non-selection of risk factor inclusion in donor screening criteria. Additional items related to TB to consider during donor review are also discussed.

References:

1. Greenaway C, Sandoe A, Vissandjee B, et al. Tuberculosis: evidence review for newly arriving immigrants and refugees. *Can Med Assoc J*. 2011;183(12):E939-E951. doi:10.1503/cmaj.090302

2. Reul NK, Gray Z, Braid BB, Leland MA. Tuberculosis Screening in Silica-Exposed Workers. *Public Health Reports*. 2022;137(2):244-254. doi:10.1177/00333549211041584

3. Rose C, Heinzerling A, Patel K, et al. Severe Silicosis in Engineered Stone Fabrication Workers — California, Colorado, Texas, and Washington, 2017–2019. *MMWR Morb Mortal Wkly Rep*. 2019;68(38):813-818. doi:10.15585/mmwr.mm6838a1

4. Nasrullah M, Mazurek JM, Wood JM, Bang KM, Kreiss K. Silicosis Mortality With Respiratory Tuberculosis in the United States, 1968-2006. *Am J Epidemiol*. 2011;174(7):839-848. doi:10.1093/aje/kwr159
